# *Lactobacillus delbrueckii* ssp. *lactis* and ssp. *bulgaricus*: a chronicle of evolution in action

**DOI:** 10.1186/1471-2164-15-407

**Published:** 2014-05-28

**Authors:** Hela El Kafsi, Johan Binesse, Valentin Loux, Julien Buratti, Samira Boudebbouze, Rozenn Dervyn, Sean Kennedy, Nathalie Galleron, Benoît Quinquis, Jean-Michel Batto, Bouziane Moumen, Emmanuelle Maguin, Maarten van de Guchte

**Affiliations:** UMR1319 Micalis, INRA, Jouy en Josas, France; UMR Micalis, AgroParisTech, Jouy en Josas, France; UR1077 Mathématique Informatique et Génome, INRA, Jouy en Josas, France; US1367 Metagenopolis, INRA, Jouy en Josas, France

**Keywords:** *Lactobacillus delbrueckii*, *Bulgaricus*, *Lactis*, Genome, Comparative genomics, Adaptation, Evolution

## Abstract

**Background:**

*Lactobacillus delbrueckii* ssp. *lactis* and ssp. *bulgaricus* are lactic acid producing bacteria that are largely used in dairy industries, notably in cheese-making and yogurt production. An earlier in-depth study of the first completely sequenced ssp. *bulgaricus* genome revealed the characteristics of a genome in an active phase of rapid evolution, in what appears to be an adaptation to the milk environment. Here we examine for the first time if the same conclusions apply to the ssp. *lactis*, and discuss intra- and inter-subspecies genomic diversity in the context of evolutionary adaptation.

**Results:**

Both *L. delbrueckii* ssp. show the signs of reductive evolution through the elimination of superfluous genes, thereby limiting their carbohydrate metabolic capacities and amino acid biosynthesis potential. In the ssp. *lactis* this reductive evolution has gone less far than in the ssp. *bulgaricus.* Consequently, the ssp. *lactis* retained more extended carbohydrate metabolizing capabilities than the ssp. *bulgaricus* but, due to high intra-subspecies diversity, very few carbohydrate substrates, if any, allow a reliable distinction of the two ssp. We further show that one of the most important traits, lactose fermentation, of one of the economically most important dairy bacteria, *L. delbruecki* ssp. *bulgaricus*, relies on horizontally acquired rather than deep ancestral genes. In this sense this bacterium may thus be regarded as a natural GMO *avant la lettre*.

**Conclusions:**

The dairy lactic acid producing bacteria *L. delbrueckii* ssp. *lactis* and ssp. *bulgaricus* appear to represent different points on the same evolutionary track of adaptation to the milk environment through the loss of superfluous functions and the acquisition of functions that allow an optimized utilization of milk resources, where the ssp. *bulgaricus* has progressed further away from the common ancestor.

**Electronic supplementary material:**

The online version of this article (doi:10.1186/1471-2164-15-407) contains supplementary material, which is available to authorized users.

## Background

The thermophilic lactic acid producing bacterium *Lactobacillus delbrueckii* has a long history of application in dairy fermentations where the subspecies (ssp.) *bulgaricus* is mainly known for its use in yogurt making while the ssp. *lactis* is traditionally used in the production of Parmesan and Emmental-type cheeses. The ssp. *bulgaricus* and *lactis* are historically distinguished on the basis of their capacity to metabolize different carbohydrates [1]. At the molecular level, a difference in the 16s rRNA sequence has been documented [2], and strains have been classified as either of the ssp. using Multi Locus Sequence Typing (MLST) [3]. The molecular basis for differing carbohydrate metabolism phenotypes has only in some cases been elucidated [4, 5].

The first complete genome sequence of *L. delbrueckii* ssp. *bulgaricus* (*L. bulgaricus*) revealed an ongoing adaptation to the protein-rich milk environment through the loss of superfluous amino acid biosynthesis functions, many of which are still recognizable in the form of pseudogenes while others have completely disappeared [6]. The nature of a number of other pseudogenes, remnants of genes involved in the transport and metabolism of various carbohydrates, pointed to an ancestor that most probably evolved in an environment where plant-carbohydrates were readily available. Evolutionary adaptation to the milk environment appears coherent with traditional yogurt-making practice, which involved the sequential transfer of samples of yogurt cultures to fresh milk. With the first records of yogurt (kisim) dating to 3200 before Christ [7], one would logically predict that *L. bulgaricus* adapted to this environment over time [6].

The ssp. *lactis* which phenotypically can be distinguished from the ssp. *bulgaricus* by its more extensive carbohydrate metabolizing capabilities, notably including the fermentation of various sugars of vegetal origin like maltose, mannose, saccharose, and trehalose [1], is less studied at the genomic level. We therefore sequenced the genomes of 6 *L. delbrueckii* strains and here present an analysis of these sequences and 4 earlier established genome sequences, together representing 5 ssp*. bulgaricus* strains and 5 ssp*. lactis* strains, to further explore the differences between the two subspecies of this economically important bacterium, notably regarding their metabolic capacities and adaptation to the milk environment.

## Results

### *L. delbrueckii*ssp. *bulgaricus*genomes are smaller than ssp. *lactis*genomes

We sequenced the genomes of 6 *L. delbrueckii* strains to near completion: 4 strains belonging to the ssp. *lactis*, and two strains from the ssp. *bulgaricus* (Table 1). The complete genome sequences of 4 other strains classified as *L. delbrueckii* ssp. *bulgaricus* (ATCC 11842 [6], ATCC BAA-365 [8], 2038 [9], NDO2 [10]) were retrieved from Genbank. An analysis of the 16S rRNA gene sequences suggested that one of these strains, NDO2, was misclassified and in fact does not belong to the ssp. *bulgaricus* as its 16S sequence does not contain the characteristic two *Eco*RI sites [2]. This conclusion was corroborated by the results of an analysis of inter-strain relationships using MLST (Additional file 1: Figure S1) where strain NDO2 does not make part of the well-defined ssp. *bulgaricus* cluster but instead appears to belong to the ssp. *lactis* or the ssp. *delbrueckii.* An alignment of the 16S rRNA sequence of strain NDO2 with the sequences of the ssp. *lactis* and ssp. *bulgaricus* strains used in this study and the sequence of the *L. delbrueckii* ssp. *delbrueckii* type strain ATCC 9649 [5] revealed 6 positions in which strain NDO2 shared nucleotides conserved in the ssp. *lactis* and *bulgaricus* strains that differ from the ssp. *delbrueckii,* and 3 positions in which strain NDO2 shared nucleotides conserved in the ssp. *lactis* and *delbrueckii* strains that differ from the ssp. *bulgaricus* (Additional file 2: Table S1). Taking the results of the different analyses together, we consider strain NDO2 as a representative of the ssp. *lactis*.

**Table 1:** **Characteristics of** ***L. delbrueckii*** **ssp.** ***bulgaricus*** **and** ***L. delbrueckii*** **ssp** ***. lactis*** **genomes**

	*L. delbrueckii*ssp*. bulgaricus*strains					*L. delbrueckii*ssp. *lactis*strains				
	ATCC 11842	ATCC BAA-365	2038	VIB27	VIB44	NDO2	CNRZ226	CNRZ327 (e)	CNRZ333	CNRZ700
Assembled genome size (a)	1,864,998	1,856,951	1,872,918	1,838,091	1,810,332	2,125,753	1,904,440	1,844,879 *1,938,538*	1,996,651	1,989,632
Estimated genome size (b)	N/A	N/A	N/A	1,853,000	1,818,000	N/A	1,911,000	1,969,000 *2,105,000*	2,052,000	2,086,000
Number of contigs	1	1	1	32	27	1	21	161/571*	87	333
Number of scaffolds	1	1	1	14	14	1	10	33/*1*	23	75
Average sequencing depth	-	-	-	86	94	-	71	78	77	56
Number of CDS (c)	1,466	1,380	1,333	1,783	1,711	1,666	1,665	1,525	1,721	1,593
Number of pseudogene-fragments (d)	630	341	459	388	423	346	390	545	381	408
Number of CDS with unknown function	642	294	343	442	434	317	361	315	369	345
Overall GC content (%)	49.7	49.7	49.7	49.4	49.7	49.6	49.8	49.8	48.2	49.5
GC content of CDS	50.8	51.2	51.9	51.7	51.8	51.5	52.0	52.2	51.6	51.8
GC content of CDS at codon position 3 (%)	65.0	64.8	64.9	66.0	66.7	64.0	67.0	65.1	63.4	67.4
CDS as % of genome sequence	73.4	68.3	69.2	77.1	76.5	75	77.4	62.9	*75.3*	*68.4*
Number of rrn operons	9	9	9	-	-	9	-	9	-	-
**Protein localization prediction**										
Cytoplasmic	1,089	996	958	1,346	1,277	1,245	1,237	1,140	1,272	1,182
Membrane	225	227	208	247	253	242	248	223	259	237
Surface exposed	86	101	115	118	115	119	119	101	123	113
Secreted	69	56	52	72	66	60	61	61	67	61

a, without paired end sequencing results.

b, assembled sequence plus estimated size of sequence gaps (estimations on the basis of paired end sequencing results).

c, not counting pseudogenes.

d, corresponding to CDS annotated as “fragment”.

e, numbers in italics represent data after genome finishing.

*The increase in the number of contigs after genome finishing is due to the addition of sequence fragments in the original sequence gaps.

-, Data not available.

Among the genomes that had earlier been sequenced to completion, the three ssp. *bulgaricus* genomes (strains ATCC 11842, ATCC BAA-365 and 2038) are smaller (1,857 to 1,873 kbp) than the ssp. *lactis* NDO2 genome (2,126 kbp) (Table 1). This observation appears to be corroborated by the estimated genome sizes of the newly sequenced strains (estimations taking assembled sequence and gap size estimations from paired end sequencing into account), which tend to be smaller for the spp. *bulgaricus* than for the ssp. *lactis* (Table 1).

An in-depth analysis of one of the newly sequenced genomes, of *L. delbrueckii* ssp. *lactis* CNRZ327, learned that virtually all remaining gaps in the sequence assembly were due to the presence of one or more repeated elements (IS elements, ribosomal RNA operons) at these sites (El Kafsi H, Binesse J, Loux V, Buratti J, Boudebbouze S, Dervyn R, Hammani A, Maguin E, van de Guchte M: Genome sequence of *Lactobacillus delbrueckii* ssp. *lactis* CNRZ327, a dairy bacterium with anti-inflammatory properties. *submitted*). Assuming that the same explanation holds for the other genomes which were sequenced to comparable depths (Table 1), we conclude that except for repeated elements the genome sequences are practically complete and allow a comparison of the predicted proteomes (see below).

### *L. delbrueckii*ssp. *lactis*genomes contain more IS elements

The finishing and detailed analysis of the *L. delbrueckii* ssp. *lactis* CNRZ327 genome revealed the presence of an extremely high number of IS elements (Table 2): 215 IS elements were detected of which 178 could be attributed to known families using IS Finder (https://www-is.biotoul.fr) [11]. The earlier published complete *L. delbrueckii* ssp. *lactis* NDO2 genome [10] also appears to contain far more (133) IS elements than the complete genomes of *L. delbrueckii* ssp. *bulgaricus* strains ATCC 11842, ATCC BAA-365, and 2038 (56, 29, and 54, respectively [6, 8, 9]) (Additional file 3: Table S2). If the number of remaining gaps in the newly sequenced genomes of the present study is an indication for the number of IS elements, as it proved to be in the case of strain CNRZ327, the same tendency is found in these genomes. Except for *L. delbrueckii* ssp. *lactis* CNRZ226, the ssp. *lactis* genomes contain significantly more IS elements than the ssp. *bulgaricus* genomes, even if the number of IS elements can also vary considerably between strains of the same subspecies. Differences in numbers of IS elements thus appear to contribute significantly to the differences in genome sizes between ssp. *bulgaricus* and ssp. *lactis* strains.

**Table 2:** **IS families in** ***L. delbrueckii*** **ssp.** ***bulgaricus*** **and** ***L. delbrueckii*** **ssp.** ***lactis***

IS family	*L. delbrueckii*ssp*. bulgaricus*ATCC 11842	*L. delbrueckii*ssp*. lactis*CNRZ327
IS30	16	57
IS 256	0	45
ISL3	2	33
IS110	8	17
IS3	1	16
IS4	12	9
ISL30	0	1
Other	17	37
Total	56	215

Numbers indicate the frequency of occurrence of different IS elements in *L. delbrueckii* ssp. *bulgaricus* ATCC 11842 [6] and *L. delbrueckii* ssp. *lactis* CNRZ327 (this study) genomes.

When comparing *L. delbrueckii* ssp. *lactis* CNRZ327 and *L. delbrueckii* ssp. *bulgaricus* ATCC 11842, striking differences are observed in the numbers of IS elements of the IS256, IS30 and ISL3 families, which are all largely overrepresented in strain CNRZ327 (Table 2). The ubiquitous IS256 family (45 copies in strain CNRZ327) is even completely absent from *L. delbrueckii* ssp. *bulgaricus* ATCC 11842 and 2038 (not shown), but it can be found in the *L. delbrueckii* ssp. *bulgaricus* ATCC BAA-365 genome (2 copies; not shown). In *L. delbrueckii* ssp. *lactis* NDO2, 7 copies are present (not shown). These observations illustrate that not only the global numbers of IS but also the numbers of IS elements of a given family largely vary between strains, including within the same ssp. No ssp*. lactis* or ssp. *bulgaricus* specific IS elements were found.

**Figure 1:**
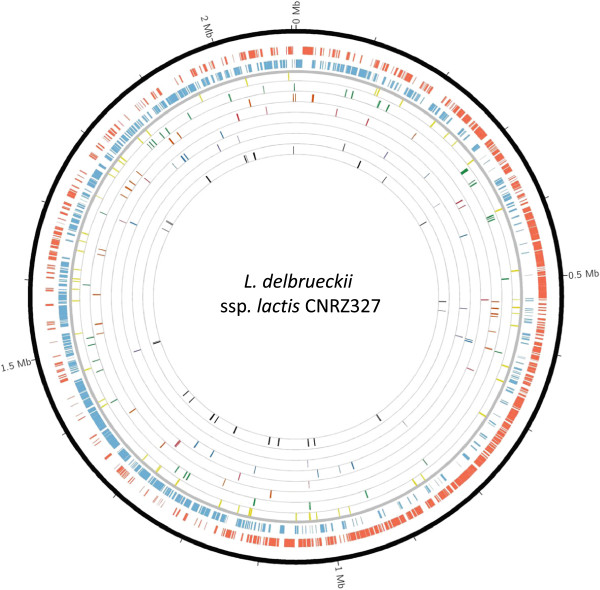
**Genome atlas of** ***L. delbrueckii*** **ssp.** ***lactis*** **CNRZ327.** The ten circles (outer to inner) show: Circles 1 and 2, CDS (excluding pseudogenes and transposases) on positive (red) or negative (blue) strand; Circle 3, transposases of the IS30 family (yellow); Circle 4, transposases of the IS256 family (green); Circle 5, transposases of the ISL3 family (orange); Circle 6, transposases of the IS110 family (red); Circle 7, transposases of the IS3 family (blue); Circle 8, transposases of the IS4 family (purple); Circle 9, transposases of ISL30 family (black) Circle 10, transposases of unkown family (grey).

In contrast with *L. bulgaricus* ATCC 11842 where a region of 415 kbp (22% of the genome) is exempt of IS elements [6] *,* in *L. delbrueckii* ssp. *lactis* CNRZ327 the IS elements seem more or less randomly distributed over the genome although a region with relatively few IS elements can be distinguished between positions 620 kb and 920 kb (Figure 1). Several locations around the genome, situated in between scaffolds of the original sequence assembly, appear to be insertion hotspots where varying combinations of two or three different IS elements are found in close proximity or in a nested configuration (not shown). 4 cases of nested IS were observed: IS256 or IS110 inserted in ISs of unknown family, IS 256 in ISL3, and IS110 in IS30. IS elements in CNRZ327 are nearly exclusively found in intergenic regions (not shown), and thus do not appear to have played an important role in the shaping of the functional gene repertoire of the ssp.

### Gene repertoires and metabolic capacities

Both the spp*. bulgaricus* and the ssp. *lactis* show distinct signs of genomes in an active state of evolution. An aberrant GC content at the third codon positions in coding sequences, a sign of rapid ongoing evolution first observed in the *L. delbrueckii* ssp. *bulgaricus* ATCC 11842 genome [6], is also observed in the other ssp. *bulgaricus* strains, as well as in the ssp*. lactis* strains (Table 1). High numbers of pseudogenes, another sign of rapid evolution, are also observed in both ssp. (Table 1). In *L. delbrueckii* ssp. *bulgaricus* strain ATCC 11842 the occurrence of pseudogenes appeared to reflect an adaptation to the protein-rich milk environment through the elimination of superfluous genes, notably involved in amino acid biosynthesis and carbohydrate metabolism [6]. In *L. delbrueckii* ssp*. lactis* CNRZ327 as well as in the other *L. delbrueckii* strains studied here, we observed a same tendency of elimination of genes involved in amino acid biosynthesis and carbohydrate metabolism (see below). In both ssp., the spontaneous deterioration of genes thus appears to have played an important role in the shaping of the functional gene repertoire of the species, through the inactivation of superfluous genes in an apparent adaptation to the environment.

**Figure 2:**
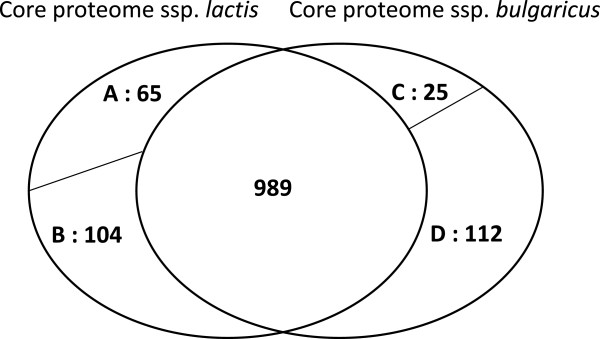
**Core proteomes of** ***L. delbrueckii*** **ssp.** ***lactis*** **and ssp.** ***bulgaricus*** **.** Ovals represent the core proteomes of *L. delbrueckii* ssp*. lactis* and *L. delbrueckii* ssp. *bulgaricus*. The overall core of the 5 ssp*. lactis* and the 5 ssp. *bulgaricus* strains in this study consists of 989 proteins. A, 65 proteins are present in all ssp*. lactis* strains and absent from all 5 ssp. *bulgaricus* strains; B, 104 proteins are present in all ssp. *lactis* strains and absent from 1 to 4 ssp. *bulgaricus* strains; C, 25 proteins are present in all ssp*. bulgaricus* strains and absent from all 5 ssp. *lactis* strains; D, 112 proteins are present in all ssp. *bulgaricus* strains and absent from 1 to 4 ssp. *lactis* strains.

In order to compare the remaining gene repertoires and metabolic capacities of the spp. *bulgaricus* and the spp. *lactis* we used two *in silico* methods and experimentally evaluated their potential of using different mono- and di-saccharides. First, we performed a clustering of orthologous proteins in order to determine the extent of the ssp. *bulgaricus* core proteome, the ssp. *lactis* core proteome, and the common core proteome of the two ssp. While one might have expected that the sizes of the respective core proteomes would reflect the earlier mentioned difference in genome size between the two ssp., this appears not to be the case: the results presented in Figure 2 show that the two ssp. have similarly sized core proteomes, which largely overlap.

When comparing the proteins from the core proteome of the ssp. *bulgaricus* that are lacking from all 5 ssp. *lactis* strains at the one hand, and the proteins from the core proteome of the ssp. *lactis* that are lacking from all 5 ssp. *bulgaricus* strains at the other (Figure 2; Additional file 4: Table S3 and Additional file 5: Table S4), it is striking that the majority (17 out of 25) of the ssp. *bulgaricus* specific proteins have no known function while the ssp. *lactis* specific proteins, which are more than twice as many, nearly all (49 out of 65) have a known (general or detailed) function. The latter proteins are mainly involved in carbohydrate and amino-acid metabolism (see below). The 8 ssp. *bulgaricus* specific proteins to which a function could be attributed are mainly involved in membrane transport. For 24 among the 65 ssp. *lactis* specific proteins, fragments of coding sequences (pseudogenes) can be found in one or more of the ssp. *bulgaricus* strains. Likewise, for 5 of the 25 ssp. *bulgaricus* specific proteins gene fragments can be found in one or more of the ssp. *lactis* strains. These observations suggest that at least part of the subspecies specificity results from the differential loss of ancestral genes.

**Figure 3:**
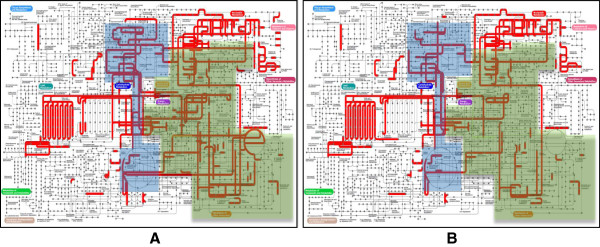
**Metabolic capacities of** ***L. delbrueckii*** **ssp.** ***lactis*** **CNRZ327 and** ***L. delbrueckii*** **ssp.** ***bulgaricus*** **ATCC 11842.** Metabolic pathway analysis was performed using KEGG [12, 13]. Graphs were generated using ipath [14]. **A**, *L. delbrueckii* ssp. *lactis* CNRZ327; **B**, *L. delbrueckii* ssp. *bulgaricus* ATCC 11842; red, enzyme functions identified in the respective genomes; highlighted in blue, carbohydrate metabolism pathways; highlighted in green, amino acid biosynthesis pathways.

A second *in silico* comparison was made using KEGG [12] functional annotations as a starting point. For this purpose, proteins from the predicted proteomes of strains ssp. *bulgaricus* ATCC 11842 and ssp. *lactis* CNRZ327 were assigned to KEGG ortholog groups and mapped to KEGG pathways using the KEGG Automatic Annotation Server (KAAS) [13]. Matching maps were compared, and for a selection of differences between the two strains the comparison was extended to the 10 strains used in this study using homology search based analyses. Not unexpectedly, the results of this analysis to a great extent corroborate the results of the core genomes analysis for as far as genes with known functions are concerned, and in addition put a certain number of differences in a metabolic pathway context. A first impression of the global metabolic capacities of the two strains obtained using Ipath [14] suggests that these capacities are more extensive for the ssp*. lactis* than for the ssp*. bulgaricus* (Figure 3), notably regarding carbohydrate and amino acid metabolism which will be discussed in more detail in the following sections together with some other features of relevance for dairy industrial bacteria.

### Carbohydrate metabolism potential is much more reduced in the ssp. *bulgaricus*than in the ssp. *lactis*

An overview of the experimentally determined carbohydrate metabolism potential of four ssp. *lactis* and four ssp. *bulgaricus* strains (Table 3) confirms that the former are capable of metabolizing a more extensive range of mono- and di-saccharides, mostly of plant origin, as has been reported earlier. However, a high level of variability was observed within the ssp. *lactis*, both with regard to the number (from 6 to 12) and the nature of the carbohydrates that could be fermented by a given strain. The ssp. *bulgaricus* strains fermented from 2 to 4 different carbohydrates. Because of this intra ssp. variability, and in contrast with the earlier description of the ssp. [1], only one of the 14 substrates for which fermentation was observed allowed to distinguish the two ssp. in our set of eight strains: N-acetylglucosamine was fermented by the ssp. *lactis* but not by the ssp. *bulgaricus*.

**Table 3:** **Carbohydrate fermentation profiles of** ***L. delbrueckii*** **strains**

	Gal	Glu	Fru	Man	N-ac	Amy	Arb	Esc	Sal	Cel	Mal	Lac	Suc	Tre	N
*L. delbrueckii* ssp. *lactis* CNRZ226	-	+	+	+	+	+	+	+	+	+	+	-	+	+	12
*L. delbrueckii* ssp*. lactis* CNRZ327	-	+	+	+	+	-	-	+	-	-	-	+	-	-	6
*L. delbrueckii* ssp*. lactis* CNRZ333	+	+	+	+	+	-	+	+	+	-	+	+	+	+	12
*L. delbrueckii* ssp*. lactis* CNRZ700	+	+	-	+	+	-	-	+	-	-	-	+	-	+	7
*L. delbrueckii* ssp*. bulgaricus* ATCC 11842	-	+	-	+	-	-	-	-	-	-	-	+	-	-	3
*L. delbrueckii* ssp*. bulgaricus* ATCC BAA-365	-	-	-	+	-	-	-	-	-	-	-	+	-	-	2
*L. delbrueckii* ssp*. bulgaricus* VIB27	-	+	-	+	-	-	-	+	-	-	-	+	-	-	4
*L. delbrueckii* ssp*. bulgaricus* VIB44	-	-	-	+	-	-	-	-	-	-	-	+	-	-	2

Carbohydrate fermentation profiles were experimentally determined using API 50 CH (Biomérieux). +, fermentation observed; -, no fermentation observed. Gal, Galactose; Glu, D-glucose; Fru, D-fructose; Man, D-mannose; N-ac, N-acetylglucosamin; Amy, Amygdalin; Arb, Arbutin; Esc, Esculin; Sal, Salicilin; Cel, Cellobiose; Mal, Maltose; Lac, Lactose; Suc, Sucrose; Tre, Trehalose; N, number of different carbohydrates fermented. Results were reproduced in two independent experiments.

With only few exceptions, the observed fermentation profiles could be predicted on the basis of *in silico* metabolic pathway analyses using existing and newly established genome sequences (Additional file 6: Table S5). The results of these *in silico* analyses also suggested that the ssp*. lactis*, in contrast to the ssp*. bulgaricus,* would be able to degrade starch (Additional file 6: Table S5). This prediction was experimentally confirmed by growth on M17 medium containing starch as the only carbon source and a starch degradation assay (results not shown).

Apart from the difference in carbohydrate metabolism potential between the two ssp., the *in silico* analyses revealed that when a carbohydrate metabolic pathway is inactive in the ssp. *lactis* this is mostly due to the lack or fragmentation of one or two pathway-specific genes while in several cases in the ssp. *bulgaricus* the same pathway is completely lacking or only represented by pseudogenes. This observation suggests that the ssp. *bulgaricus* represents a more advanced state of elimination of ancestral carbohydrate metabolic pathways than the ssp*. lactis*.

The results of the *in silico* analysis also indicate that for the uptake of carbohydrates *L. delbrueckii* mostly relies on active transport using PTS systems or ABC transporters (Additional file 6: Table S5). A noticeable exception is the uptake of the milk sugar lactose in the ssp. *bulgaricus*, which will be described below.

### Lactose metabolism in the ssp. *bulgaricus*relies on horizontally acquired genes

**Figure 4:**
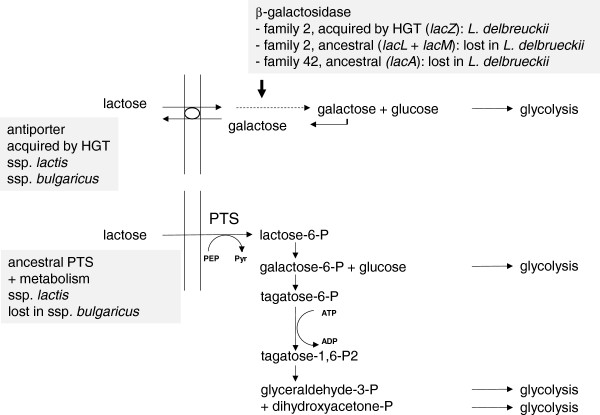
**Lactose transport and metabolism pathways in** ***L. delbrueckii*** **and its ancestors.** In *L. delbrueckii* ssp. *bulgaricus*, lactose uptake relies on a lactose-galactose antiporter which has been acquired by horizontal gene transfer while the ancestral lactose PTS system has been lost. *L. delbrueckii* ssp. *lactis* contains both transport systems. In *L. delbrueckii* ssp. *bulgaricus*, lactose metabolism relies on a β-galactosidase which has been acquired by horizontal gene transfer while the ancestral β-galactosidases have been lost. *L. delbrueckii* ssp. *lactis* contains the same horizontally acquired β-galactosidase and, in addition, the pathways to completely metabolize lactose-6-P generated by the lactose PTS-system. PEP, phosphoenolpyruvate; Pyr, pyruvate.

The comparative genomics approach using KEGG pathways led to some particularly interesting observations with regard to the uptake and metabolism of the milk sugar lactose, a key feature of dairy lactic acid bacteria. While for the ssp. *bulgaricus* it is well established that lactose enters the cell via an antiporter which, after cleavage of lactose by a β-galactosidase, extrudes the non-metabolizable galactose moiety of the molecule [4, 15, 16], it appears that nearly all the the ssp. *lactis* strains in addition possess a lactose PTS system to import and phosphorylate lactose, a phospho-β-galactosidase to cleave the lactose-6-P formed, and the enzymes needed to metabolize the galactose-6-phosphate liberated in the latter reaction (Additional file 6: Table S5). Using this system, the spp. *lactis* would thus be able to metabolize not only the glucose moiety of lactose, like the spp. *bulgaricus*, but also the galactose moiety (Figure 4).

This PTS system, the phospho-β-galactosidase and the galactose-6-P metabolizing enzymes appear to be rare in the lactobacilli of the acidophilus group and in lactobacilli in general. The phylogeny of the lactose specific PTS system component (LacE) and the phospho-β-galactosidase (LacG) appears to be coherent with the 16S rRNA phylogeny (Additional file 7: Figure S2), however, suggesting that they are encoded by ancestral genes which have been lost in most lactobacilli, including *L. delbrueckii* ssp. *bulgaricus*. The first signs of a loss of this system are also found in the ssp. *lactis,* where in strain CNRZ226 the whole system is missing, like in the ssp. *bulgaricus* (Additional file 6: Table S5).

In contrast, several lines of evidence suggest that the lactose-galactose antiporter permease and the functionally associated β-galactosidase have most probably been acquired by horizontal gene transfer in the ancestral *L. delbrueckii* lineage: 1) while some of the closely related bacteria in the *Lactobacillus acidophilus* group contain a β-galactosidase (belonging to the glycoside hydrolase family 2) encoded in two genes (*lacL* and *lacM,* for large and small subunit, respectively), the homologous family 2 β-galactosidase of *L. delbrueckii* is of the type that resembles that found in *Streptococcus*, encoded in one gene (*lacZ*) (Additional file 8: Figure S3) and 2) the lactose-galactose antiporter (lactose permease, encoded by *lacS*) of *L. delbrueckii* shows a higher resemblance to its *Streptococcus* homologues than to its homologues in closely related lactobacilli, causing a disagreement between the LacS-based phylogeny and 16S rRNA-based phylogeny indicative of horizontal transfer between the *L. delbrueckii* lineage and the *Streptococcus* lineage (Additional file 8: Figure S3). It is interesting to note that several lactobacilli of the acidophilus group (*L. johnsonii, L. acidophilus*, *L. crispatus, L. helveticus*) and some other lactobacilli in addition to the two-gene family 2 β-galactosidase contain LacA, a β-galactosidase of the glycoside hydrolase family 42, which also appears to be ancestral (Additional file 9: Figure S4), showing the same phylogeny as the 16S rRNA genes. This family 42 protein is not present in *L. delbrueckii*.

It thus appears that, in spite of the presence of ancestral genes encoding a lactose permease, β-galactosidases belonging to two different glycoside hydrolase families, a lactose PTS, and a phospho-β-galactosidase in the *L. acidophilus* group, the lactose fermenting capacity of one of the most important dairy bacteria in this group, *L. delbrueckii* ssp. *bulgaricus*, relies on horizontally acquired rather than deep ancestral genes. As earlier reported [5], the *lac* repressor is inactivated in the ssp*. bulgaricus* strains. Inactivation of this repressor and the loss of the galactose metabolic pathway in the spp. *bulgaricus* have been presented as possibly having contributed to the selection of this ssp. for fast fermentation of milk where lactose is abundant [5, 17]. Furthermore, the ancestral PTS-lactose system still visible in the ssp. *lactis* suggests that the ancestor has its origin in a low lactose environment, where the PTS system would ensure a level of efficiency of lactose transport and energy harvesting that the antiporter system cannot provide.

### Amino acid biosynthesis capacities are more severely reduced in the ssp. *bulgaricus*than in the ssp. *lactis*

A second key to the comprehension of *L. delbrueckii*’s metabolic capacities is the protein richness of the milk environment. Earlier studies of the *L. bulgaricus* ATCC 11842 genome revealed a highly reduced capacity of amino acid biosynthesis which may be explained as an adaptation to this environment where the bacterium’s proteolytic system can liberate amino acids from the abundant milk proteins, thus rendering amino acid biosynthesis pathways superfluous [6]. This view is confirmed in the other *L. bulgaricus* strains in this study (Additional file 10: Table S6). In the ssp. *lactis*, the same tendency can be observed albeit in a less advanced state: fewer amino acid biosynthesis pathways are inactivated (Additional file 10: Table S6), in non-functional pathways less genes are inactivated (Additional file 10: Table S6), and pseudo-genes are less fragmented than in the *ssp. bulgaricus* (not shown). All strains of the ssp. *lactis* are predicted to synthesize lysine, aspartate and arginine, while most of the ssp*. bulgaricus* strains cannot synthesize these amino-acids (Additional file 10: Table S6). Neither of the two ssp. would be able to produce phenylalanine, tyrosine, tryptophan, glycine, serine, alanine, glutamate, valine, leucine, isoleucine or histidine.

Both ssp. contain an important proteolytic system with about 30 proteins annotated as proteinases or peptidases (not shown). This number is not different, however, from the number found in *Lactobacillus gasseri*, a closely related *Lactobacillus acidophilus* group member from the gastro-intestinal tract (not shown). The only significant adaptation of the proteolytic system of both *L. delbrueckii* ssp. *bulgaricus* and ssp. *lactis* appears to be the presence of the major cell wall bound protease PrtB, which is responsible for milk casein degradation [18]. This protease is not found in other lactobacilli of the acidophilus group except *Lactobacillus equicursoris*, the closest known relative of *L. delbrueckii*.

### Less genes involved in acid tolerance in the ssp. *bulgaricus*than in the ssp. *lactis*

*L. delbrueckii* acidifies its growth medium through the production of lactate, and acid tolerance therefore is an important aspect of its physiology. In spite of this, the analysis of the ssp. *bulgaricus* ATCC 11842 genome revealed the presence of only few of the diverse systems that have been implicated in acid tolerance in lactic acid bacteria [19], and suggested that an H + transporting ATPase constitutes its primary defense, with a possible contribution of cation:proton antiporters and two ornithine decarboxylases [6]. For the latter enzymes, *L. bulgaricus* appears to rely on ornithine produced by *Streptococcus thermophilus*, its companion bacterium in yogurt fermentation [6]. This vision is corroborated by the analysis of the other ssp. *bulgaricus* strains in the present study (Additional file 11: Table S7). The ssp. *lactis* genomes in contrast reveal a more diverse set of genes involved in acid stress resistance where, in addition to the functions present in the spp. *bulgaricus*, ornithine as well as pH lowering ammonia and CO_2_ can be produced through the arginine deiminase pathway (encoded by the *arc*A, *arc*B and *arc*C genes). Ornithine can then be used to import more arginine through an arginine-ornithine antiporter (encoded by *arc*D), or be decarboxylated by ornithine decarboxylase for which the ssp. *lactis* strains possess one gene copy. In one of the ssp. *lactis* strains, CNRZ700, *arc*A appears to be a pseudogene, while the same is true for the *arc*D gene in strains CNRZ327 and NDO2 (Additional file 11: Table S7). In the ssp. *bulgaricus* strains, *arc*D is present as a pseudogene, while no traces are found of the *arc*A, B, or C genes.

The ssp*. lactis* genomes also contain remnants of another gene that has been implicated in acid tolerance, encoding a glutamate decarboxylase, of which no trace can be found in the ssp. *bulgaricus* strains. Both the arginine deiminase pathway and glutamate decarboxylase can be found in other lactobacilli of the acidophilus group, and the above mentioned observations suggest that *L. delbrueckii* ssp. *bulgaricus* has lost these functions while the ssp. *lactis* is following the same way.

## Discussion

*Lactobacillus delbrueckii* is one of the economically most important bacteria in dairy industries, where the ssp. *bulgaricus* is mainly known from its application in yogurt fermentation and the ssp. *lactis* from its use in the production of Parmesan and Emmenthal type cheeses. The classical procedure of distinguishing the two ssp. on the basis of their phenotypical features, in particular their carbohydrate fermentation profiles, has clearly shown its limits, as again confirmed in this study. While the ssp. *lactis* strains in this study were on an average capable of fermenting a larger pallet of (up to 12) carbohydrates than the ssp. *bulgaricus* strains, a high level of variability was observed between strains of the same ssp. and only one monosaccharide substrate was identified that allowed to reliably discriminate the two ssp., even among the small number of strains studied.

The *in silico* analysis of the first complete genome sequence of an *L. bulgaricus* strain [6] indicated that this strain derived from an ancestor that would have been capable of fermenting a number of carbohydrates of plant origin, and suggested that *L. bulgaricus* adapted to the milk environment by reductive evolution in which superfluous metabolic pathways went through a process of gene inactivation and elimination. Here, we extend this observation to four other *L. bulgaricus* genomes and demonstrate that the differential loss of genes involved in carbohydrate metabolism in different strains is at the basis of the observed variability in fermentation capacities. For the ssp. *bulgaricus* strain ATCC BAA-365, adaptation to the milk environment has gone so far that it only metabolizes one sugar, mannose, other than the milk sugar lactose. In this study we extend these genome-scale observations for the first time to the ssp*. lactis*, where in four newly established and one existing genome sequence a similar, but less advanced, adaptation to the milk medium can be recognized. Intriguingly, while for any of 14 mono- and di-saccharides studied at least one and on an average 4 out of 8 strains lost the capacity to ferment it, all *L. delbrueckii* ssp. *bulgaricus* and ssp. *lactis* strains studied preserved their capacity to ferment the plant sugar mannose.

A second major adaptation to the milk environment concerns the loss of amino acid biosynthesis pathways, earlier observed in *L. bulgaricus* [6] and here also in the ssp. *lactis*. Like in the case of carbohydrate metabolism, this adaptation appears to be less advanced in the ssp. *lactis* than in the ssp. *bulgaricus*. Explained by the ample availability of amino acids in the form of milk proteins, this adaptation is coherent with the presence of the major cell wall bound protease PrtB responsible for the first step in the degradation of milk proteins in both ssp. The gene encoding this proteinase is not found in closely related lactobacilli. From these examples, the general picture emerges that both *L. delbrueckii* ssp. are in the course of adaptation to the protein and lactose rich milk environment, where the ssp. *bulgaricus* has advanced further in this direction. This conclusion is in line with an earlier study by Germond et al. [5] who postulated on the basis of 16S rRNA analysis that the spp. *bulgaricus* had evolved further away from the common ancestor than the ssp. *lactis*. The common ancestor would have evolved in an environment where various carbohydrates from plant origin constituted the main carbon sources, as can be deduced from the presence of (remnants of) genes involved in their fermentation in the various strains analyzed.

Interestingly, our analyses indicate that while the ssp. *bulgaricus* and *lactis* possess an acquired lactose-galactose antiporter to import the milk sugar lactose, the common ancestor possessed a dedicated PTS system, which is still present in the ssp*. lactis,* for this purpose. Whereas an antiporter is the system of choice in the lactose-rich milk environment, a PTS system, which excels in conditions where the substrate concentration is low, points to an ancestral environment where lactose was present but in low concentrations. Together with the indication that the ancestral environment contained carbohydrates from plant origin, this may suggest that the ancestor evolved in the mammalian digestive tract, an environment where both conditions can be met. This hypothesis is coherent with the fact that most of the known closely related lactobacilli of the acidophilus group are gut isolates, and further backed by the presence of genes coding for putative mucus binding proteins in 4 of the 5 ssp. *lactis* genomes analyzed (not shown).

With genomes that similar which represent different stages of adaptation to the milk environment, what is the difference between the ssp. *lactis* and *bulgaricus* that is important to dairy industries? Earlier reports mentioned the capacity of faster and more reliable milk fermentation by the ssp. *bulgaricus* than by the ssp*. lactis*, which was explained by the inactivation of the *lac* repressor in the former resulting in the constitutive expression of β-galactosidase, the enzyme that cleaves lactose to yield glucose and galactose [20]. Likewise, the protease prtB has been reported to be constitutively expressed in *L. bulgaricus*, while being tightly regulated in the ssp*. lactis* [21]. These examples indicate that at least part of the industrially relevant difference between the two ssp. may be found in gene regulation rather than in gene content. High intra ssp. variability makes it difficult to pinpoint further industrially relevant features, and in view of the evolutionary picture emerging from the present study chances are high that desired properties can be found among either ssp. From a dairy industrial point of view, the distinction of the two ssp. may therefore look artificial.

## Conclusions

The dairy lactic acid producing bacteria *L. delbrueckii* ssp. *lactis* and ssp. *bulgaricus* appear to represent different points on the same evolutionary track of adaptation to the milk environment through the loss of superfluous functions, where the ssp. *bulgaricus* has progressed further away from the common ancestor. Interestingly, it appears that one of the most important traits, lactose fermentation, of one of the economically most important dairy bacteria, *L. delbruecki* ssp. *bulgaricus*, relies on horizontally acquired rather than deep ancestral genes. In this sense this bacterium may thus be regarded as a natural GMO *avant la lettre*.

## Methods

### Bacterial strains

The bacterial strains of which the genome sequences were determined in this work were obtained from the INRA collection and belong to the subspecies *L. delbrueckii* ssp. *bulgaricus* (strains Vib27 and Vib44) or *L. delbrueckii* ssp. *lactis* (strains CNRZ327, CNRZ333, CNRZ226, CNRZ700). For as far documented, the ssp. *bulgaricus* strains were originally isolated from yogurt while the ssp. *lactis* strains were derived from Emmental cheese or the starter cultures used to produce this type of cheese. Bacteria were grown at 42°C under microaerobic conditions in MRS broth (Difco) or on the same medium solidified with 2% agar.

### Genome sequencing

Genome sequences for 6 *L. delbrueckii* strains, 2 of the ssp. *bulgaricus* and 4 of the ssp. *lactis*, were generated by 454 paired-end sequencing (Roche Life Sciences) followed by sequence assembly using Newbler 2.6 (Roche). For one strain, *L. delbrueckii* ssp. *lactis* CNRZ327, the resulting scaffolds were ordered using Mauve aligner [22] with the earlier published genome sequence of *L. bulgaricus* ATCC 11842 [6] as the reference, and a finishing protocol was applied in order to evaluate the level of completeness of the sequence (El Kafsi H, Binesse J, Loux V, Buratti J, Boudebbouze S, Dervyn R, Hammani A, Maguin E, van de Guchte M: Genome sequence of *Lactobacillus delbrueckii* ssp. *lactis* CNRZ327, a dairy bacterium with anti-inflammatory properties. *submitted*). Genome sequences were deposited in the European Nucleotide Archive (http://www.ebi.ac.uk/ena) under the accession numbers CCDV01000001 (strain CNRZ327), CCDS01000001-CCDS01000023 (strain CNRZ333), CCDT01000001-CCDT01000010 (strain CNRZ226), CCDU01000001-CCDU01000075 (strain CNRZ700), CCET01000001-CCET01000014 (strain VIB27), and CCEU01000001-CCEU01000014 (strain VIB44). Earlier published complete genome sequences were retrieved from NCBI [GenBxank:CR954253.1 (strain ATCC 11842), GenBank:CP000412.1 (strain ATCC BAA-365), GenBank:CP000156.1 (strain 2038), GenBank:CP002341.1 (strain NDO2)].

### Genome annotation

Genome annotation was performed using AGMIAL [23], preferably transferring annotations from the *L. bulgaricus* ATCC11842 annotation [6] where appropriate. Insertion sequences were attributed to IS families using ISfinder (https://www-is.biotoul.fr) [11]. Pseudogenes were detected by aligning predicted CDS against Uniprot release 2013_06 using blastx. In case of CDS aligning with longer or smaller proteins in the database (±10% threshold), the database protein was back-aligned to the genome sequence with tblastn. When the resulting alignment was longer than the original CDS, with the presence of a frameshift or a stop codon in the genome sequence, the original predicted CDS was marked as pseudogene-fragment.

### KEGG pathway annotation

Proteins from strains ATCC 11842 and CNRZ327 were assigned to KEGG (Kyoto Encyclopedia of Genes and Genomes) ortholog groups and mapped to KEGG pathways using the KEGG Automatic Annotation Server (KAAS, http://www.genome.jp/kegg/kaas/) [13] with parameters “BBH method” and genes data set “eco, bsu, sau, lmo, lla, spy, spn, ste, lpl, lpj, ljo, ljf, lac, lsa, lsl, ldb, lbu, lbr, lca, lcb, lga, lre, lrf, lhe, lfe, lrh, lrl”. The results were visualized using KEGG or ipath (http://pathways.embl.de/) [14].

### Protein clustering

In order to determine the core proteomes of *L. delbrueckii* and the two ssp. studied, protein sequences encoded by non-pseudogenes in the different genomes were compared using blastp to define groups of orthologous proteins by single linkage clustering (e-value < 10^−3^; > 78% identity over > 76% of the longest sequence length).

### Multi Locus Sequence Typing

MLST analysis was performed with MEGA5 [24] as described in [3], using the conserved parts of 7 housekeeping genes (*fusA, gyrB, hsp60, ileS, pyrG, recA, recG*) and including the strains with known *L. delbrueckii* subspecies attribution studied in [3].

### Phylogenetic construction

MEGA5 [24] was used to construct strain phylogenetic trees (1000 bootstrap replications). Trees were drawn using jplot [25].

### Carbohydrate fermentation profiling

Carbohydrate fermentation profiles of the strains in this study were established using API 50 CH (Biomérieux) according to the instructions of the supplier, with readout after 48 hr of incubation at 42°C. The capacity to metabolize starch was evaluated by the ability to grow on M17 medium (Difco) solidified with 2% agar and containing 1% starch (Merck, analytical grade) as the only carbon source. Alternatively, bacteria were grown on MRS medium (Difco) solidified with 2% agar and containing 1% starch, after which starch degradation was visualized through coloration of remaining starch by exposure to iodine.

## Electronic supplementary material

Additional file 1: Figure S1: Phylogenetic analysis of *L. delbrueckii* strains using MLST. The phylogenetic tree was constructed using MEGA software [24]. *, strains used in the present study; (a) originally classified as *L. delbrueckii* ssp. *bulgaricus* in [10]. The scale bar represents the mean number of nucleotide substitutions per site. (DOC 108 KB)

Additional file 2: Table S1: *L. delbrueckii* subspecies specific nucleotides in 16S rRNA sequences. (DOC 42 KB)

Additional file 3: Table S2: Number of IS elements in *L. delbrueckii* ssp. *bulgaricus* and ssp*. lactis.* (DOCX 11 KB)

Additional file 4: Table S3: *L. delbrueckii* ssp. *bulgaricus* specific proteins. (DOC 48 KB)

Additional file 5: Table S4: *L. delbrueckii* ssp. *lactis* specific proteins. (DOC 80 KB)

Additional file 6: Table S5: Genes involved in carbohydrate metabolism in *L. delbrueckii* ssp. *lactis* and ssp. *bulgaricus* strains. (DOC 166 KB)

Additional file 7: Figure S2: Coherence between 16S rRNA-based phylogeny and *lacE* and *lacG* based phylogenies. Alignment of nucleotide (16S rRNA) or protein (*LacE, LacG*) sequences and tree construction were performed using ClustalW [26], and trees were drawn using njplot [25]. A, lactose specific PTS system component (*lacE*) phylogeny; B, phospho-β-galactosidase (*lacG*) phylogeny; C, 16S rRNA phylogeny. Numbers indicate bootstrap values; the scale bar represents the mean number of nucleotide or amino acid substitutions per site. (DOC 60 KB)

Additional file 8: Figure S3: Inconsistency between 16S rRNA-based phylogeny and *lacS* and β-galactosidase based phylogenies. Alignment of nucleotide (16S rRNA) or protein (β-galactosidase) sequences and tree construction were performed using ClustalW [26], and trees were drawn using njplot [25]. A, lactose permease (*lacS*) phylogeny; B, β-galactosidase (*lacL* or *lacZ*) phylogeny; C, 16S rRNA phylogeny. *, β-galactosidase large subunit (*lacL*), belonging to the glycoside hydrolase family 2; **, homologous family 2 β-galactosidase (*LacZ*) encoded in one gene. Numbers indicate bootstrap values; the scale bar represents the mean number of nucleotide or amino acid substitutions per site. (DOC 74 KB)

Additional file 9: Figure S4: Coherence between 16S rRNA-based phylogeny and *lacA* based phylogeny. Alignment of nucleotide (16S rRNA) or protein (*LacA*) sequences and tree construction were performed using ClustalW [26], and trees were drawn using njplot [25]. A, family 42 β-galactosidase (*lacA*) phylogeny; B, 16S rRNA phylogeny. Numbers indicate bootstrap values; the scale bar represents the mean number of nucleotide or amino acid substitutions per site. (DOC 50 KB)

Additional file 10: Table S6: Genes involved in amino acid metabolism in *L. delbrueckii* ssp. *lactis* and ssp. *bulgaricus* strains. (DOC 206 KB)

Additional file 11: Table S7: Genes involved in acid stress resistance. (DOC 54 KB)
