# Supplementary figures and images for: Lactobacillus delbrueckii ssp. lactis and ssp. bulgaricus: a chronicle of evolution in action
Source: BMC Genomics. 2014 May 28;15(1):407. doi: 10.1186/1471-2164-15-407 (PMC4082628; doi:10.1186/1471-2164-15-407)

Additional file 1: Figure S1


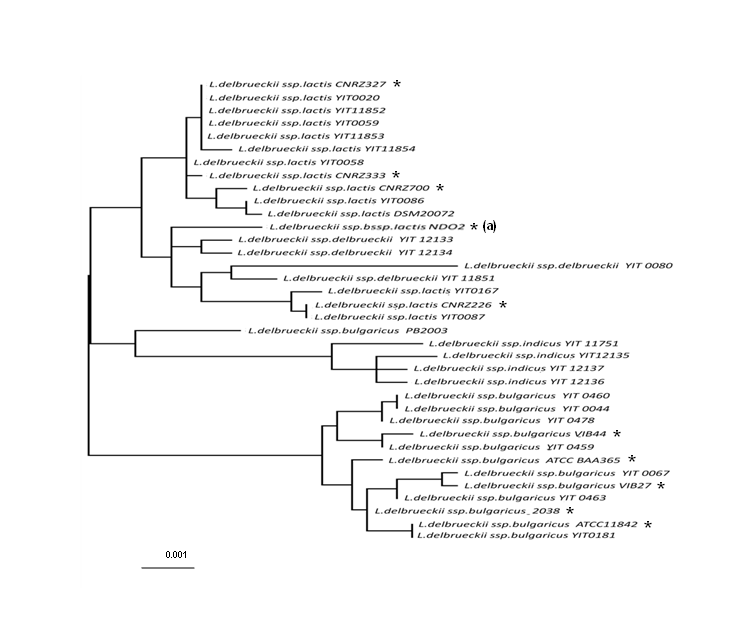

Supplement: Supplementary file 1 — Additional file 1: Figure S1: Phylogenetic analysis of L. delbrueckii strains using MLST. The phylogenetic tree was constructed using MEGA software [24]. *, strains used in the present study; (a) originally classified as L. delbrueckii ssp. bulgaricus in [10]. The scale bar represents the mean number of nucleotide substitutions per site. (DOC 108 KB) [file 12864_2014_6193_MOESM1_ESM.doc]
