# Supplementary material for: Lactobacillus delbrueckii ssp. lactis and ssp. bulgaricus: a chronicle of evolution in action
Source: BMC Genomics. 2014 May 28;15(1):407. doi: 10.1186/1471-2164-15-407 (PMC4082628; doi:10.1186/1471-2164-15-407)
Supplement: Supplementary file 2 — Additional file 2: Table S1: L. delbrueckii subspecies specific nucleotides in 16S rRNA sequences. (DOC 42 KB) [file 12864_2014_6193_MOESM2_ESM.doc]

**Add 2: Table S1. *L. delbrueckii* subspecies specific nucleotides in 16S rRNA sequences.**

| Strain | Nucleotide position | | | | | | | | |
| --- | --- | --- | --- | --- | --- | --- | --- | --- | --- |
|  | 7 | 9 | 21 | 224 | 432 | 690* | 859 | 1273 | 1455 |
| *L. delbrueckii* ssp*. bulgaricus ATCC 11842* | T | A | G | T | T | T | G | G | G |
| *L. delbrueckii* ssp*. bulgaricus ATCC BAA-365* | T | A | G | T | T | T | G | G | G |
| *L. delbrueckii* ssp*. bulgaricus 2038* | T | A | G | T | T | T | G | G | G |
| *L. delbrueckii* ssp*. bulgaricus VIB27* | T | A | G | T | T | T | G | G | G |
| *L. delbrueckii* ssp*. bulgaricus VIB44* | T | A | G | T | T | T | G | G | G |
| *L. delbrueckii* ssp*. lactis NDO2* (a) | T | A | G | C | C | C | G | G | G |
| *L. delbrueckii* ssp*. lactis CNRZ226* | T | A | G | C | C | C | G | G | - |
| *L. delbrueckii* ssp*. lactis CNRZ327* | T | A | G | C | C | C | G | G | G |
| *L. delbrueckii* ssp*. Lactis CNRZ333* | T | A | G | C | C | C | G | G | G |
| *L. delbrueckii* ssp*. lactis CNRZ700* | T | A | G | C | C | C | G | G | G |
| *L. delbrueckii* ssp*. delbrueckii ATCC 9649* | A | C | - | C | C | C | A | A | A |

16S rRNA sequences from *L. delbrueckii* strains belonging to the ssp *bulgaricus, lactis*, and *delbrueckii* were aligned using Clustalw [26]. Only differences that allow to distinguish subspecies are shown. Nucleotide position, position in the ATCC 11842 sequence.

*Mutation generating an *Eco*RI site in the *L. delbrueckii* ssp. *bulgaricus* 16S rDNA. a, originally classified as ssp. *bulgaricus* .
