# Supplementary material for: Lactobacillus delbrueckii ssp. lactis and ssp. bulgaricus: a chronicle of evolution in action
Source: BMC Genomics. 2014 May 28;15(1):407. doi: 10.1186/1471-2164-15-407 (PMC4082628; doi:10.1186/1471-2164-15-407)
Supplement: Supplementary file 3 — Additional file 3: Table S2: Number of IS elements in L. delbrueckii ssp. bulgaricus and ssp. lactis. (DOCX 11 KB) [file 12864_2014_6193_MOESM3_ESM.docx]

**Add 3: Table S2. Number of IS elements in *L. delbrueckii* ssp*. bulgaricus* and ssp*. lactis*.**

| Strain | Number of IS | Genome size (Mbp) |
| --- | --- | --- |
| *Lactobacillus delbrueckii* ssp*. bulgaricus ATCC 11842* | 56 | 1,86 |
| *Lactobacillus delbrueckii* ssp*. bulgaricus ATCC BAA-365* | 29 | 1,85 |
| *Lactobacillus delbrueckii* ssp*. bulgaricus 2038* | 54 | 1.87 |
| *Lactobacillus delbrueckii* ssp*. lactis ND02* | 133 | 2,12 |
| *Lactobacillus delbrueckii* ssp*. lactis CNRZ327* | 215 | 2,11 |
