# Supplementary material for: Lactobacillus delbrueckii ssp. lactis and ssp. bulgaricus: a chronicle of evolution in action
Source: BMC Genomics. 2014 May 28;15(1):407. doi: 10.1186/1471-2164-15-407 (PMC4082628; doi:10.1186/1471-2164-15-407)
Supplement: Supplementary file 4 — Additional file 4: Table S3: L. delbrueckii ssp. bulgaricus specific proteins. (DOC 48 KB) [file 12864_2014_6193_MOESM4_ESM.doc]

**Add 4: Table S3. *L. delbrueckii* ssp. *bulgaricus* specific proteins.**

| **Locus_tag** | **Protein** | **Function** |
| --- | --- | --- |
| Ldb_1456 | Putative permease | Transport/binding protein and lipoproteins |
| Ldb_0014 | ABC transporter, ATP-binding/permease protein | Transport/binding protein and lipoproteins |
| Ldb_1891 | Permease | Ttransport/binding protein and lipoproteins |
| Ldb_2094 | ABC transporter, ATP-binding/permease protein | Transport/binding protein and lipoproteins |
| Ldb_0502 | Putative permease | Transport/binding protein and lipoproteins |
| Ldb_1775 | Ornithine decarboxylase | Metabolism of amino acids and related molecules |
| Ldb_0262 | Pyridoxine 5 -phosphate oxidase V related favin-nucleotide-binding protein | Pyridoxal phosphate biosynthetis |
| Ldb_1782 | Transposase | Transposon and IS |
| Ldb_1287 | Hypothetical protein | ND |
| Ldb_1296 | Hypothetical membrane protein | ND |
| Ldb_0158 | Hypothetical protein | ND |
| Ldb_1547 | Hypothetical protein | ND |
| Ldb_1548 | Hypothetical protein | ND |
| Ldb_1547 | Hypothetical protein | ND |
| Ldb_1682 | Hypothetical protein | ND |
| Ldb_1743 | Hypothetical protein | ND |
| Ldb_1766 | Hypothetical membrane protein | ND |
| Ldb_1781 | Conserved hypothetical membrane protein | ND |
| Ldb_1797 | Hypothetical protein | ND |
| Ldb_0061 | Hypothetical protein | ND |
| Ldb_0890 | Hypothetical protein | ND |
| Ldb_0313 | Hypothetical protein | ND |
| Ldb_0074 | Hypothetical protein | ND |
| Ldb_0921 | Hypothetical protein | ND |
| Ldb_0915 | Hypothetical protein | ND |

Presented proteins make part of the core proteome of the ssp. *bulgaricus* (5 strains), and are lacking from all 5 ssp. *lactis* strains in this study. Locus tag, identifier of the corresponding protein in *L. bulgaricus* ATCC 11842 . ND, not determined.
