# Supplementary material for: Lactobacillus delbrueckii ssp. lactis and ssp. bulgaricus: a chronicle of evolution in action
Source: BMC Genomics. 2014 May 28;15(1):407. doi: 10.1186/1471-2164-15-407 (PMC4082628; doi:10.1186/1471-2164-15-407)
Supplement: Supplementary file 5 — Additional file 5: Table S4: L. delbrueckii ssp. lactis specific proteins. (DOC 80 KB) [file 12864_2014_6193_MOESM5_ESM.doc]

**Add 5: Table S4. *L. delbrueckii* ssp. *lactis* specific proteins.**

| **Locus_tag** | **Protein** | **Function** |
| --- | --- | --- |
| LBCNRZ327_03570 | ABC superfamily ATP binding cassette transporter, ABC protein | Transporter / binding protein |
| LBCNRZ327_02370 | Oligopeptide binding protein | Transporter / binding protein |
| LBCNRZ327_00535 | ABC transporter substrate binding protein | Transporter / binding protein |
| LBCNRZ327_01470 | Oligopeptide ABC superfamily ATP binding cassette transporter, binding protein | Transporter / binding protein |
| LBCNRZ327_02360 | Periplasmic oligopeptide-binding family protein | Transporter / binding protein |
| LBCNRZ327_02715 | Maltose ABC superfamily ATP binding cassette transporter, permease protein | Transporter / binding protein of carbohydrates |
| LBCNRZ327_02725 | Sugar ABC superfamily ATP binding cassette transporter, ABC protein | Transporter / binding protein of carbohydrates |
| LBCNRZ327_07155 | Mannitol PTS, EIIA | Transporter / binding protein of carbohydrates |
| LBCNRZ327_02730 | ß-phosphoglucomutase | Metabolism of carbohydrates and related molecules |
| LBCNRZ327_08295 | Phosphoenolpyruvate synthase | Metabolism of carbohydrates and related molecules |
| LBCNRZ327_03650 | Phosphoglycerate mutase | Transport and metabolism of carbohydrates |
| LBCNRZ327_02735 | Maltose phosphorylase | Metabolism of carbohydrates and related molecules |
| LBCNRZ327_05415 | Peptidoglycan-binding protein | Metabolism of carbohydrates and related molecules |
| LBCNRZ327_08780 | Ribose-5-phosphate isomerase B | Metabolism of carbohydrates and related molecules |
| LBCNRZ327_05435 | Phospho-ß-glycosidase | Metabolism of carbohydrates and related molecules |
| LBCNRZ327_07165 | Mannitol-1-phosphate 5-dehydrogenase | Metabolism of carbohydrates and related molecules |
| LBCNRZ327_02750 | LacI family transcriptional regulator | Metabolism of carbohydrates and related molecules |
| LBCNRZ327_03615 | Carbamoyl-phosphate synthase large chain 2 | Metabolism of amino acids and related molecules |
| LBCNRZ327_09715 | Ornithine carbamoyltransferase | Metabolism of amino acids and related molecules |
| LBCNRZ327_09720 | Carbamate kinase | Metabolism of amino acids and related molecules |
| LBCNRZ327_09725 | Arginine deiminase | Metabolism of amino acids and related molecules |
| LBCNRZ327_08995 | 3-dehydroquinate dehydratase | Metabolism of amino acids and related molecules |
| LBCNRZ327_03960 | Argininosuccinate synthase | Metabolism of amino acids and related molecules |
| LBCNRZ327_04140 | Glutamate 5-kinase | Metabolism of amino acids and related molecules |
| LBCNRZ327_04145 | Gamma-glutamyl phosphate reductase | Metabolism of amino acids and related molecules |
| LBCNRZ327_04855 | NAD-dependent aldehyde dehydrogenase | Metabolism of amino acids and related molecules |
| LBCNRZ327_05000 | Glutamine ABC superfamily ATP binding cassette transporter, permease protein | Metabolism of amino acids and related molecules |
| LBCNRZ327_06445 | Dipeptidase A | Metabolism of amino acids and related molecules |
| LBCNRZ327_08630 | Pyrroline-5-carboxylate reductase | Metabolism of amino acids and related molecules |
| LBCNRZ327_05430 | D-serine/D-alanine/glycine:H+ symporter | Transport/ binding of amino-acids |
| LBCNRZ327_05805 | HNH endonuclease domain protein | Metabolism of nucleotides and nucleic acids |
| LBCNRZ327_05810 | Dihydroorotase | Metabolism of nucleotides and nucleic acids |
| LBCNRZ327_08930 | Aspartate carbamoyltransferase | Metabolism of nucleotides and nucleic acids |
| LBCNRZ327_03965 | Acyl-CoA dehydrogenase | Lipid transport and metabolism |
| LBCNRZ327_03980 | Transcriptional regulator (LysR family) | Transcription regulation |
| LBCNRZ327_05400 | LysR substrate binding domain protein | Transcription regulation |
| LBCNRZ327_03570 | Putative repressor LexA | Transcription regulation |
| LBCNRZ327_07145 | Mannitol operon transcriptional antiterminator | Transcription regulation |
| LBCNRZ327_09025 | LysR substrate binding domain protein | Transcription regulation |
| LBCNRZ327_06440 | Signal recognition particle-docking protein FtsY | Protein secretion |
| LBCNRZ327_06170 | Possible (S)-2-hydroxy-acid oxidase | Glyoxylate and dicarboxylate metabolism |
| LBCNRZ327_00415 | Transposase | Transposon and IS |
| LBCNRZ327_00575 | Acetyltransferase, GNAT family | [N-acetyltransferase activity](http://www.ebi.ac.uk/QuickGO/GTerm?id=GO:0008080) |
| LBCNRZ327_00695 | Signal transduction diguanylate cyclase | [phosphorus-oxygen lyase activity](http://www.ebi.ac.uk/QuickGO/GTerm?id=GO:0016849) |
| LBCNRZ327_02595 | Hemerythrin HHE cation binding domain protein | [phosphorelay sensor kinase activity](http://www.ebi.ac.uk/QuickGO/GTerm?id=GO:0000155) |
| LBCNRZ327_02600 | N-6 adenine-specific DNA methylase YitW | site-specific DNA-methyltransferase (adenine-specific) activity |
| LBCNRZ327_03405 | Glutathione-disulfide reductase | [flavin adenine dinucleotide binding](http://www.ebi.ac.uk/QuickGO/GTerm?id=GO:0050660) |
| LBCNRZ327_10040 | Glutathione reductase | Posttranslational modification, protein turnover, chaperones |
| LBCNRZ327_10055 | Possible ATPase involved in chromosome partitioning | Cell cycle control, cell division, chromosome partitioning |
| LBCNRZ327_10070 | DNA starvation/stationary phase protection protein Dps | Inorganic ion transport and metabolism |
| LBCNRZ327_08230 | DegV family protein | ND |
| LBCNRZ327_09545 | Conserved domain protein | ND |
| LBCNRZ327_10310 | Predicted nucleoside-diphosphate-sugar epimerase | ND |
| LBCNRZ327_10450 | Putative uncharacterized protein | ND |
| LBCNRZ327_07930 | Putative uncharacterized protein | ND |
| LBCNRZ327_05725 | Putative uncharacterized protein | ND |
| LBCNRZ327_00715 | Hypothetical protein | ND |
| LBCNRZ327_02430 | Conserved domain protein | ND |
| LBCNRZ327_03420 | Putative uncharacterized protein | ND |
| LBCNRZ327_04640 | Putative uncharacterized protein | ND |
| LBCNRZ327_04730 | Putative uncharacterized protein | ND |
| LBCNRZ327_04940 | Putative uncharacterized protein | ND |
| LBCNRZ327_05055 | Conserved domain protein | ND |
| LBCNRZ327_07360 | Putative uncharacterized protein | ND |
| LBCNRZ327_07365 | Putative uncharacterized protein | ND |

Presented proteins make part of the core proteome of the ssp. *lactis* (5 strains), and are lacking from all 5 ssp. *bulgaricus* strains in this study. Locus tag, identifier of the corresponding protein in *L. delbrueckii* ssp. *lactis* CNRZ327. ND, not determined.
