# Supplementary material for: Lactobacillus delbrueckii ssp. lactis and ssp. bulgaricus: a chronicle of evolution in action
Source: BMC Genomics. 2014 May 28;15(1):407. doi: 10.1186/1471-2164-15-407 (PMC4082628; doi:10.1186/1471-2164-15-407)
Supplement: Supplementary file 6 — Additional file 6: Table S5: Genes involved in carbohydrate metabolism in L. delbrueckii ssp. lactis and ssp. bulgaricus strains. (DOC 166 KB) [file 12864_2014_6193_MOESM6_ESM.doc]

**Add 6: Table S5. Genes involved in carbohydrate metabolism in *L. delbrueckii* ssp. *lactis* and ssp. *bulgaricus* strains.**

|  |  |  | **ssp. *lactis* strains** | | | | | **ssp. *bulgaricus* strains** | | | | |
| --- | --- | --- | --- | --- | --- | --- | --- | --- | --- | --- | --- | --- |
| EC number | Enzyme | Gene  name | CNRZ226 | CNRZ327 | CNRZ333 | CNRZ700 | NDO2 | ATCC 11842 | ATCC BAA365 | VIB27 | VIB44 | 2038 |
| **Galactose metabolism** | | | | | | | | | | | | |
| **-** | Transporter not identified | - |  |  |  |  |  |  |  |  |  |  |
| 2.7.1.6 | Galactokinase | *galK* |  |  |  | F |  |  |  |  |  |  |
| 2.7.7.12 | Galactose 1-P uridylyltransferase | *galT* |  |  |  | F |  |  |  |  |  |  |
| API profile | | | - | - | + | + | ND | - | - | - | - | ND |
| **Sucrose transport and metabolism** | | | | | | | | | | | | |
| 2.7.1.69 | PTS family sucrose porter, EIIBC component | *ScrA* |  |  |  | F |  | F | F | F | F | F |
| 3.2.1.26 | Sucrose 6P hydrolase (sucrase) | *ScrB* |  |  |  | F |  | F | F | F | F | F |
| 2.7.1.4 | Fructokinase | *ScrK* |  |  |  | F |  | F | F | F | F | F |
| API profile | | | + | - | + | - | ND | - | - | - | - | ND |
| **Maltose transport and metabolism** | | | | | | | | | | | | |
| - | Maltose ABC transporter permease protein (1) | *MalE/F/G* |  |  |  | F |  | F | F | F | F | F |
| 2.4.1.8 | Maltose phosphorylase (1) | *mapA* |  |  |  |  |  |  |  |  |  |  |
| 5.4.2.6 | ß-phosphoglucomutase (1) | *pgmA* |  |  |  |  |  |  |  |  |  |  |
| - | Maltose ABC transporter permease protein (2) | *malE/F/G* |  |  |  | F |  | F | F | F | F | F |
| 3.2.1.20 | Oligo-1,6-glucosidase (2) | *malL* |  |  |  |  |  |  |  |  |  |  |
| 2.7.1.69 | PTS –Glucose specific EIIA (3) | - |  |  |  |  |  |  |  |  |  |  |
| 3.2.1.122 | Maltose-6'-phosphate glucosidase (3) | *malH* |  | F |  |  |  |  |  |  | F |  |
| API profile | | | + | - | + | - | ND | - | - | - | - | ND |
| **Cellobiose transport and metabolism** | | | | | | | | | | | | |
| 2.7.1.69 | PTS system, cellobiose-specific IIABC component | *celA/B/C* |  | F | F | F | F | F | F | F | F | F |
| 3.2.1.86 | 6-Phospho-ß-glucosidase | *pbg3* |  |  |  | F |  |  |  |  | F |  |
| 5.3.1.9 | Glucose-6-phosphate isomerase | *-* |  |  |  |  |  |  |  |  |  |  |
| - | Transcriptional regulator | *yeca* |  |  |  |  |  |  |  |  |  |  |
| API profile | | | + | - | - | - | ND | - | - | - | - | ND |
| **Arbutin transport and metabolism** | | | | | | | | | | | | |
| 2.7.1.69 | PTS system, arbutin-specific | *-* |  |  |  | F |  |  |  |  |  |  |
| 3.2.1.86 | 6-phospho-ß-glucosidase | *pbg3* |  |  |  | F |  |  |  |  | F |  |
| 5.3.1.9 | Glucose-6-phosphate isomerase | *-* |  |  |  |  |  |  |  |  |  |  |
| API profile | | | + | - | + | - | ND | - | - | - | - | ND |
| **Salicin transport and metabolism** | | | | | | | | | | | | |
| - | Unidentified transporter | *-* |  |  |  |  |  |  |  |  |  |  |
| 3.2.1.86 | 6-Phospho-ß-glucosidase | *pbg3* |  |  |  | F |  |  |  |  | F |  |
| 5.3.1.9 | Glucose-6-phosphate isomerase | *-* |  |  |  |  |  |  |  |  |  |  |
| API profile | | | + | - | + | - | ND | - | - | - | - | ND |
| **D-glucose transport and metabolism** | | | | | | | | | | | | |
| 2.7.1.69 | PTS system, glucose/glucoside-specific enzyme | *-* |  |  |  |  |  |  |  |  |  |  |
| 5.3.1.9 | Glucose-6-phosphate isomerase | *pgi* |  |  |  |  |  |  |  |  |  |  |
| API profile | | | + | + | + | + | ND | + | - | + | - | ND |
| **Lactose transport and metabolism** | | | | | | | | | | | | |
| 2.7.1.69 | PTS family lactose porter, IIABC components (1) | *LacE/LacF* |  |  |  |  |  |  |  |  |  |  |
| - | Transcription antiterminator (1) | *LacT* |  |  |  |  |  |  |  |  |  |  |
| 3.2.1.85 | 6-Phospho-b-galactosidase (1) | *LacG* |  |  |  |  |  |  |  |  |  |  |
| 5.3.1.26 | Galactose-6-phosphate isomerase LacB subunit(1) | *LacB* |  |  |  |  |  |  |  |  |  |  |
| 5.3.1.26 | Galactose-6-phosphate isomerase LacA subunit (1) | *LacA* |  |  |  |  |  |  |  |  |  |  |
| 2.7.1.144 | Tagatose-6-phosphate kinase (1) | *LacC* |  |  |  |  |  |  |  |  |  |  |
| 4.1.2.40 | Tagatose 1,6-diphosphate aldolase (1) | *LacD* |  |  |  |  |  |  |  |  |  |  |
| - | Lactose permease (2) | *LacS* |  |  |  |  |  |  |  |  |  |  |
| 3.2.1.23 | ß-galactosidase (2) | *LacZ* |  |  |  |  |  |  |  |  |  |  |
| - | Lac repressor (2) | *LacR* | F |  |  |  |  | F | F | F | F | F |
| API profile | | | - | + | + | + | ND | + | + | + | + | ND |
| **Mannose transport and metabolism** | | | | | | | | | | | | |
| 2.7.1.69 | PTS system, mannose-specific enzyme IIABCD components | *-* |  |  |  |  |  |  |  |  |  |  |
| 5.3.1.8 | Mannose-6-phosphate isomerase | *pmi* |  |  |  |  |  |  |  |  |  |  |
| 2.7.1.11 | 6-Phosphofructokinase | *pfK2* |  |  |  |  |  |  |  |  |  |  |
| 4.1.2.13 | Fructose-bisphosphate aldolase | *fba* |  |  |  |  |  |  |  |  |  |  |
| API profile | | | + | + | + | + | ND | + | + | + | + | ND |
| **Fructose transport and metabolism** | | | | | | | | | | | | |
| 2.7.1.69 | PTS system, fructose-specific enzyme IIABC component (1) | *fruB* |  |  |  |  |  |  |  |  |  |  |
| 2.7.1.56 | 1-Phosphofructokinase (1) | *fruk* |  |  |  |  |  |  |  |  |  |  |
| - | Fructose repressor (1) | *fruR* |  |  |  |  |  |  | F |  | F |  |
| - | Unidentified Fructose permease (2) |  |  |  |  |  |  |  |  |  |  |  |
| 2.7.1.4 | Fructokinase (2) | *ScrK* |  |  |  | F |  | F | F | F | F | F |
| 2.7.1.11 | 6-Phosphofructokinase(2) | *pfk2* |  |  |  |  |  |  |  |  |  |  |
| 4.1.2.13 | Fructose-bisphosphate aldolase (2) | *fba* |  |  |  |  |  |  |  |  |  |  |
| API profile | | | + | + | + | - | ND | - | - | - | - | ND |
| **N-acetylglycosamin transport and metabolism** | | | | | | | | | | | | |
| - | Unidentified transporter | *-* |  |  |  |  |  |  |  |  |  |  |
| 3.5.1.25 | N-acetylglucosamine-6-phosphate deacetylase | *nagA* |  |  |  |  |  |  |  |  |  | F |
| 3.5.99.6 | Glucosamine-6-phosphate deaminase | *nagB* |  |  |  |  | F | F | F | F | F | F |
| API profile | | | + | + | + | + | ND | - | - | - | - | ND |
| **Trehalose transport and metabolism** | | | | | | | | | | | | |
| 2.7.1.69 | PTS family trehalose porter, IIBC component | *treB* |  |  |  |  |  |  |  |  |  |  |
| 3.2.1.93 | Trehalose-6-phosphate hydrolase | *dexS* |  |  |  |  |  |  |  |  |  |  |
|  | API profile | | + | - | + | + | ND | - | - | **-** | - | ND |
| **Esculin transport and metabolism** | | | | | | | | | | | | |
| - | Pathway not described by KEGG | - |  |  |  |  |  |  |  |  |  |  |
| API profile | | | + | + | + | + | ND | - | - | + | - | ND |
| **Amygdalin tansport and metabolism** | | | | | | | | | | | | |
| - | Pathway not described by KEGG | - |  |  |  |  |  |  |  |  |  |  |
| API profile | | | + | - | - | - | ND | - | - | - | - | ND |
| **Starch metabolism** | | | | | | | | | | | | |
| 3.2.1.135 | Neopullulanase | *nplT** |  |  |  |  |  |  |  |  |  |  |
| 3.2.1.70 | Glucan 1,6-alpha -glucosidase | *dexB** |  |  |  |  |  | F | F | F | F | F |
| Degradation profile | | | + | + | + | + | ND | - | - | - | - | ND |

Grey box, gene is present; white box, gene is absent; F, gene fragment(s) only. API profile or Degradation profile, results of experimental determination of capacity to ferment the carbohydrate indicated: +, fermentation observed; -, no fermentation observed; ND, not determined. (1), (2), (3) designate alternative pathways.

*These genes are absent from table S4 generated by single linkage protein clustering due to limitations imposed by the choice of criteria for automatic protein clustering.
