# Supplementary material for: Lactobacillus delbrueckii ssp. lactis and ssp. bulgaricus: a chronicle of evolution in action
Source: BMC Genomics. 2014 May 28;15(1):407. doi: 10.1186/1471-2164-15-407 (PMC4082628; doi:10.1186/1471-2164-15-407)

**Add 7: Figure S2. Coherence between 16S rRNA-based phylogeny and *lac*E and *lac*G based phylogenies.** Alignment of nucleotide sequences and tree construction were performed using ClustalW [26], and trees were drawn using njplot [25]. A, lactose specific PTS system component (*lacE* gene) phylogeny; B, phospho-β-galactosidase (*lacG* gene) phylogeny; C, 16S rRNA phylogeny. Numbers indicate bootstrap values; the scale bar represents the mean number of nucleotide substitutions per site.


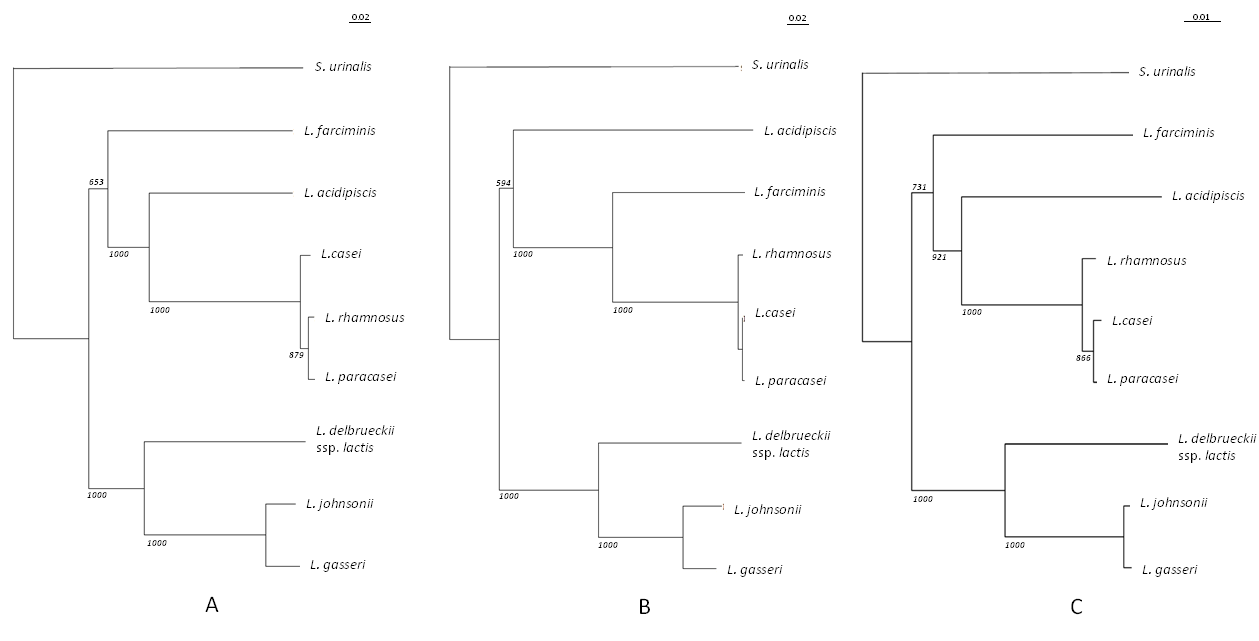

Supplement: Supplementary file 7 — Additional file 7: Figure S2: Coherence between 16S rRNA-based phylogeny and lacE and lacG based phylogenies. Alignment of nucleotide (16S rRNA) or protein (LacE, LacG) sequences and tree construction were performed using ClustalW [26], and trees were drawn using njplot [25]. A, lactose specific PTS system component (lacE) phylogeny; B, phospho-β-galactosidase (lacG) phylogeny; C, 16S rRNA phylogeny. Numbers indicate bootstrap values; the scale bar represents the mean number of nucleotide or amino acid substitutions per site. (DOC 60 KB) [file 12864_2014_6193_MOESM7_ESM.doc]
