# Supplementary material for: Lactobacillus delbrueckii ssp. lactis and ssp. bulgaricus: a chronicle of evolution in action
Source: BMC Genomics. 2014 May 28;15(1):407. doi: 10.1186/1471-2164-15-407 (PMC4082628; doi:10.1186/1471-2164-15-407)

**Add 8: Figure S3. Inconsistency between 16S rRNA-based phylogeny and *lac*S and β-galactosidase gene based phylogenies.** Alignment of nucleotide sequences and tree construction were performed using ClustalW [26], and trees were drawn using njplot [25]. A, lactose permease (*lacS* gene) phylogeny; B, β-galactosidase (*lacL* or *lacZ* gene) phylogeny; C, 16S rRNA phylogeny. *, β-galactosidase large subunit (*lacL* gene), belonging to the glycoside hydrolase family 2; **, homologous family 2 β-galactosidase encoded in one gene (*lacZ*). Numbers indicate bootstrap values; the scale bar represents the mean number of nucleotide substitutions per site.


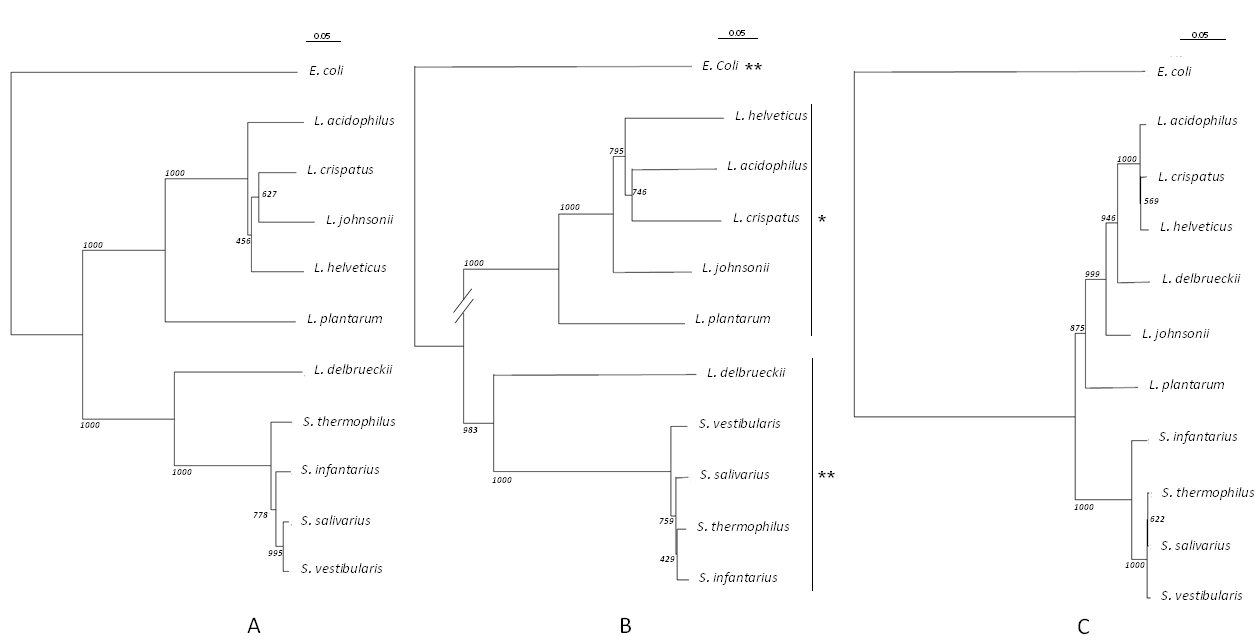

Supplement: Supplementary file 8 — Additional file 8: Figure S3: Inconsistency between 16S rRNA-based phylogeny and lacS and β-galactosidase based phylogenies. Alignment of nucleotide (16S rRNA) or protein (β-galactosidase) sequences and tree construction were performed using ClustalW [26], and trees were drawn using njplot [25]. A, lactose permease (lacS) phylogeny; B, β-galactosidase (lacL or lacZ) phylogeny; C, 16S rRNA phylogeny. *, β-galactosidase large subunit (lacL), belonging to the glycoside hydrolase family 2; **, homologous family 2 β-galactosidase (LacZ) encoded in one gene. Numbers indicate bootstrap values; the scale bar represents the mean number of nucleotide or amino acid substitutions per site. (DOC 74 KB) [file 12864_2014_6193_MOESM8_ESM.doc]
