# Supplementary material for: Lactobacillus delbrueckii ssp. lactis and ssp. bulgaricus: a chronicle of evolution in action
Source: BMC Genomics. 2014 May 28;15(1):407. doi: 10.1186/1471-2164-15-407 (PMC4082628; doi:10.1186/1471-2164-15-407)

**Add 9: Figure S4. Coherence between 16S rRNA-based phylogeny and *lacA* based phylogeny.** Alignment of nucleotide sequences and tree construction were performed using ClustalW [26], and trees were drawn using njplot [25]. A, family 42 β-galactosidase (*lacA* gene) phylogeny; B, 16S rRNA phylogeny. Numbers indicate bootstrap values; the scale bar represents the mean number of nucleotide substitutions per site.


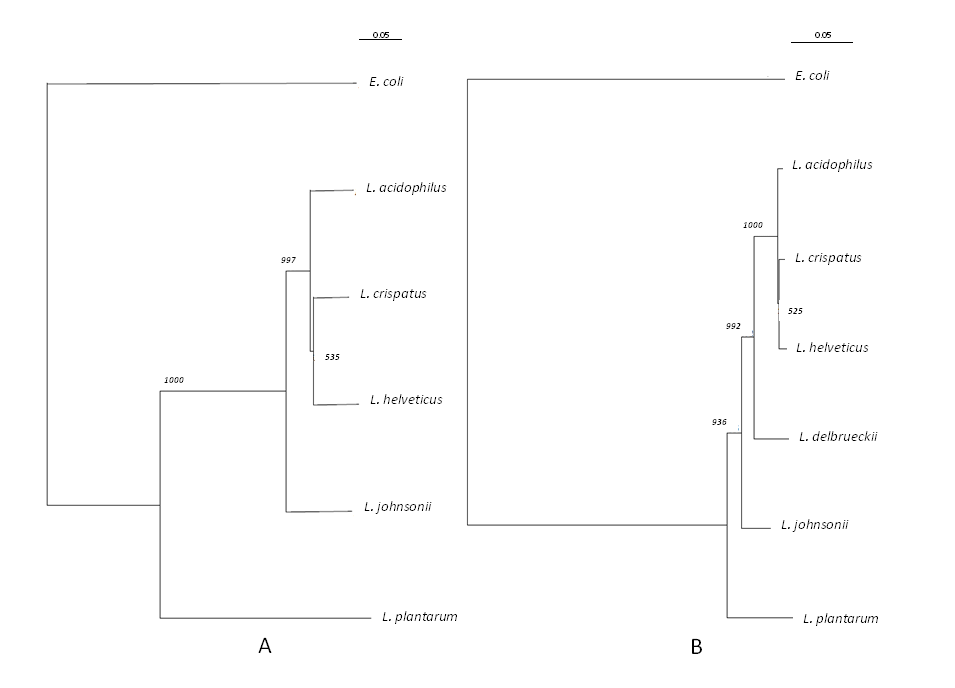

Supplement: Supplementary file 9 — Additional file 9: Figure S4: Coherence between 16S rRNA-based phylogeny and lacA based phylogeny. Alignment of nucleotide (16S rRNA) or protein (LacA) sequences and tree construction were performed using ClustalW [26], and trees were drawn using njplot [25]. A, family 42 β-galactosidase (lacA) phylogeny; B, 16S rRNA phylogeny. Numbers indicate bootstrap values; the scale bar represents the mean number of nucleotide or amino acid substitutions per site. (DOC 50 KB) [file 12864_2014_6193_MOESM9_ESM.doc]
