# Supplementary material for: Lactobacillus delbrueckii ssp. lactis and ssp. bulgaricus: a chronicle of evolution in action
Source: BMC Genomics. 2014 May 28;15(1):407. doi: 10.1186/1471-2164-15-407 (PMC4082628; doi:10.1186/1471-2164-15-407)
Supplement: Supplementary file 10 — Additional file 10: Table S6: Genes involved in amino acid metabolism in L. delbrueckii ssp. lactis and ssp. bulgaricus strains. (DOC 206 KB) [file 12864_2014_6193_MOESM10_ESM.doc]

**Add 10: Table S6. Genes involved in amino acid metabolism in *L. delbrueckii* ssp. *lactis* and ssp. *bulgaricus*** strains.

|  |  | |  | | **ssp. *lactis* strains** | | | | | **ssp. *bulgaricus* strains** | | | | | | |
| --- | --- | --- | --- | --- | --- | --- | --- | --- | --- | --- | --- | --- | --- | --- | --- | --- |
| EC number | Enzyme | | Gene name | | CNRZ226 | CNRZ327 | CNRZ333 | CNRZ700 | NDO2 | ATCC 11842 | ATCC BAA365 | VIB27 | | VIB44 | | 2038 |
| **Threonine biosynthesis** | | | | | | | | | | | | | | | | |
| 2.7.2.4 | Aspartate kinase | | *ask* | |  |  |  |  |  |  |  |  |  | |  | |
| 1.2.1.11 | Aspartate-semialdehyde dehydrogenase | | *-* | |  |  |  |  |  |  |  |  |  | |  | |
| 1.1.1.3 | Homoserine dehydrogenase | | *thrA* | | F |  |  |  |  |  |  |  |  | |  | |
| 2.7.1.39 | Homoserine kinase | | *thrB* | |  |  |  |  |  |  |  |  |  | |  | |
| 4.2.3.1 | Homoserine synthase | | *thrC* | |  |  |  |  |  |  |  |  |  | |  | |
| **Lysine biosynthesis** | | | | | | | | | | | | | | | | |
| 2.7.2.4 | Aspartokinase | | | *lysC* |  |  |  |  |  |  |  |  |  | |  | |
| 1.2.1.11 | Aspartate semialdehyde dehydrogenase | | | *asd* |  |  |  |  |  |  |  |  |  | |  | |
| 4.3.3.7 | Dihydrodipicolinate synthase | | | *dapA* |  |  |  |  |  |  |  |  |  | |  | |
| 1.3.1.26 | Dihydrodipicolinate reductase | | | *dapB* |  |  |  |  |  |  |  |  |  | |  | |
| 2.3.1.89 | 2,3,4,5 Tetrahydropyridine-2,6-dicarboxylate N-acetyl-transferase | | | *dapD* |  |  |  |  |  |  |  |  |  | |  | |
| 2.6.1.- | Transaminase | | | *aspC1* |  |  |  |  |  |  |  |  |  | |  | |
| 3.5.1.47 | N-acetyldiaminopimelate deacetylase | | | *hipO* |  |  |  |  |  |  |  |  |  | |  | |
| 5.1.1.7 | Diamino pimelate epimerase | | | *dapF* |  |  |  |  |  |  |  |  |  | |  | |
| 4.1.1.20 | Diamino pimelate decarboxylase | | | *lysA* |  |  |  |  |  |  |  |  |  | |  | |
| **Arginine biosynthesis** | | | | | | | | | | | | | | | | |
| 6.3.1.2 | Glutamine synthetase | | *glnA* | |  |  |  |  |  |  |  |  |  | |  | |
| 2.7.2.2 | Carbamate kinase | | *arcC* | |  |  |  |  |  |  |  |  |  | |  | |
| 2.1.3.3 | Ornithine carbamoyl transferase | | *argF* | |  |  |  |  |  |  |  |  |  | |  | |
| 6.3.4.5 | Arginino succinate synthase | | *argG* | |  |  |  |  |  |  |  |  |  | |  | |
| 4.3.2.1 | Arginino succinate lyase | | *argH* | |  |  |  |  |  | F | F | F | F | | F | |
| 3.5.3.6 | Arginine deiminase | | *arcA* | |  |  |  |  |  |  |  |  |  | |  | |
| **Aspartic acid biosynthesis** | | | | | | | | | | | | | | | | |
| 4.3.1.1 | Aspartate ammonia-lyase | | *aspA* | |  |  |  |  |  |  |  |  |  | |  | |
| 6.3.5.4 | Asparagine synthetase | | *asnB* | |  |  |  |  |  |  |  |  |  | |  | |
| 2.6.1.1 | Aspartate aminotransferase | | *aspC1* | |  |  |  |  |  |  |  |  |  | |  | |
| **Proline biosynthesis** | | | | | | | | | | | | | | | | |
| 2.7.2.11 | Glutamate 5-kinase | | *proB* | |  | F |  |  |  | F | F | F | F | | F | |
| 1.2.1.41 | Gamma-glutamyl phosphate reductase | | *proA* | |  |  |  |  |  |  |  |  |  | |  | |
| 1.5.1.2 | Pyroline-5-carboxylate reductase | | *proC* | |  |  |  |  |  |  |  |  |  | |  | |
| **Serine biosynthesis** | | | | | | | | | | | | | | | | |
| 1.1.1.95 | Phosphoglycerate dehydrogenase | | *serA* | |  |  |  |  |  |  |  |  |  | |  | |
| 2.6.1.52 | Phosphosérine aminotransferase | | *serC* | |  |  |  |  |  |  |  |  |  | |  | |
| 3.1.3.3 | Phosphoserine phosphatase | | *serB* | |  |  |  |  |  |  |  |  |  | |  | |
| **Tryptophan biosynthesis** | | | | | | | | | | | | | | | | |
| 1.1.1.95 | Phosphoglycerate dehydrogenase | | serA | |  |  |  |  |  |  |  |  |  | |  | |
| 2.6.1.52 | Phosphosérine aminotransferase | | *serC* | |  |  |  |  |  |  |  |  |  | |  | |
| 3.1.3.3 | Phosphoserine phosphatase | | *serB* | |  |  |  |  |  |  |  |  |  | |  | |
| 4.2.1.20 | Tryptophan synthase ß-chain | | *trpB* | |  |  |  |  |  |  |  |  |  | |  | |
| **Tyrosine biosynthesis** | | | | | | | | | | | | | | | | |
| 4.2.1.10 | 3-Dehydroquinate dehydratase | | *aroD* | |  |  |  |  |  |  |  |  |  | |  | |
| 1.1.1.25 | Shikimate dehydrogenase | | *aroE* | | F |  | F | F | F |  |  |  |  | |  | |
| 2.7.1.71 | Shikimate Kinase | | *aroK* | | F | F | F | F | F | F | F | F | F | | F | |
| 2.5.1.19 | 3-Phosphoshikimate 1-carboxyvinyl transferase | | *aroA* | |  |  |  |  | F | F | F | F | F | | F | |
| 4.2.3.5 | Chorismate synthase | | *aroC* | |  |  |  |  |  | F | F | F | F | |  | |
| 5.4.99.5 | Chorismate mutase | | *pheB* | |  |  |  |  |  |  |  |  |  | |  | |
| 1.3.1.12 | Prephenate dehydrogenase | | *-* | |  |  |  |  |  |  |  |  |  | |  | |
| 2.6.1.57 | Aromatic amino acid aminotransferase | | *-* | |  |  |  |  |  |  |  |  |  | |  | |
| **Phenylalanine biosynthesis** | | | | | | | | | | | | | | | | |
| 4.2.1.10 | 3-Dehydroquinate dehydratase | | *aroD* | |  |  |  |  |  |  |  |  |  | |  | |
| 1.1.1.25 | Shikimate dehydrogenase | | *aroE* | | F |  | F | F | F |  |  |  |  | |  | |
| 2.7.1.71 | Shikimate Kinase | | *aroK* | | F | F | F | F | F | F | F | F | F | | F | |
| 2.5.1.19 | 3-Phosphoshikimate 1-carboxyvinyl transferase | | *aroA* | |  |  |  |  | F | F | F | F | F | | F | |
| 4.2.3.5 | Chorismate synthase | | *aroC* | |  |  |  |  |  | F | F | F | F | |  | |
| 5.4.99.5 | Chorismate mutase | | *pheB* | |  |  |  |  |  |  |  |  |  | |  | |
| 4.2.1.51 | Prephenate dehydratase | | *-* | |  |  |  |  |  |  |  |  |  | |  | |
| 2.6.1.57 | Aromatic amino acid aminotransferase | | - | |  |  |  |  |  |  |  |  |  | |  | |
| **Cysteine biosynthesis** | | | | | | | | | | | | | | | | |
| 2.3.1.30 | Serine O-acetyltransferase | | *cysE* | |  |  |  |  |  | F | F | F | F | | F | |
| 2.5.1.49 | Cysteine synthase | | *cysK* | |  |  |  |  |  |  |  |  |  | |  | |
| **Methionine biosynthesis** | | | | | | | | | | | | | | | | |
| 2.3.1.30 | Serine O-acetyltransferase | | *cysE* | |  |  |  |  |  | F | F | F | F | | F | |
| 2.5.1.49 | Cysteine synthase | | *cysK* | |  |  |  |  |  |  |  |  |  | |  | |
| 4.4.1.1 | Cystathionine gamma-lyase | | *-* | |  |  |  |  |  |  |  |  |  | |  | |
| 4.4.1.8 | Cystathionine ß-lyase | | *patC* | |  |  |  |  |  |  |  |  |  | |  | |
| 2.1.1.10 | Homocysteine S-methyltransferase | | *-* | |  |  |  |  |  |  |  |  |  | |  | |
| **Glutamine biosynthesis** | | | | | | | | | | | | | | | | |
| 6.3.1.2 | Glutamine synthetase | | *glnA* | |  |  |  |  |  |  |  |  |  | |  | |
| **Alanine biosynthesis** | | | | | | | | | | | | | | | | |
| 2.6.1.21 | D-amino-acid transaminase | | *-* | |  |  |  |  |  |  |  |  |  | |  | |
| 2.6.1.41 | D-methionine—pyruvate transaminase | | *-* | |  |  |  |  |  |  |  |  |  | |  | |
| 5.1.1.1 | Alanine racemase | | *Alr* | |  |  |  |  |  |  |  |  |  | |  | |
| **Glutamic acid biosynthesis** | | | | | | | | | | | | | | | | |
| 1.4.1.2/3/4 | Glutamate dehydrogenase | | - | |  |  |  |  |  |  |  |  |  | |  | |
| 2.6.1.1 | Aspartate aminotransferase | | - | |  |  |  |  |  |  |  |  |  | |  | |
| 1.4.1.13/14 | Glutamate synthase | | - | |  |  |  |  |  |  |  |  |  | |  | |
| **Glycine biosynthesis** | | | | | | | | | | | | | | | | |
| 2.1.2.1 | Glycine hydroxymethyltransferase | | - | |  |  |  |  |  |  |  |  |  | |  | |
| 4.1.2.5 | Threonine aldolase | | - | |  |  |  |  |  |  |  |  |  | |  | |
| **Valine biosynthesis** | | | | | | | | | | | | | | | | |
| 4.1.3.18 | | Acetolactate synthase | - | |  |  |  |  |  |  |  |  |  | |  | |
| 5.4.99.3 | | 2-Acetolactate mutase | - | |  |  |  |  |  |  |  |  |  | |  | |
| 1.1.1.86 | | Ketol-acid reductoisomerase | - | |  |  |  |  |  |  |  |  |  | |  | |
| 4.2.1.9 | | Dihydroxy-acid dehydratase | - | |  |  |  |  |  |  |  |  |  | |  | |
| **Leucine biosynthesis** | | | | | | | | | | | | | | | | |
| 4.1.3.18 | | Acetolactate synthase | - | |  |  |  |  |  |  |  |  |  | |  | |
| 5.4.99.3 | | 2-Acetolactate mutase | - | |  |  |  |  |  |  |  |  |  | |  | |
| 1.1.1.86 | | Ketol-acid reductoisomerase | - | |  |  |  |  |  |  |  |  |  | |  | |
| 4.2.1.9 | | Dihydroxy-acid dehydratase | - | |  |  |  |  |  |  |  |  |  | |  | |
| **Isoleucine biosynthesis** | | | | | | | | | | | | | | | | |
| 4.3.1.19 | | Threonine ammonia-lyase | - | |  |  |  |  |  |  |  |  |  | |  | |
| 4.1.3.18 | | Acetolactate synthase | - | |  |  |  |  |  |  |  |  |  | |  | |
| 1.1.1.86 | | Ketol-acid reductoisomerase | - | |  |  |  |  |  |  |  |  |  | |  | |
| 4.2.1.9 | | Dihydroxy-acid dehydratase | - | |  |  |  |  |  |  |  |  |  | |  | |
| 2.6.1.42 | | Branched chain amino acid aminotransferase | - | |  |  |  |  |  |  |  |  |  | |  | |
| **Histidine biosynthesis** | | | | | | | | | | | | | | | | |
| 2.7.1.15 | Ribokinase | | *rbsK* | |  |  |  |  |  |  |  |  |  | |  | |
| 2.7.6.1 | Ribose-P pyrophosphokinase | | *Prs* | |  |  |  |  |  |  |  |  |  | |  | |
| 2.4.2.17 | ATP phosphoribosyltransferase | | - | |  |  |  |  |  |  |  |  |  | |  | |
| 3.6.1.31 | Phosphoribosyl-ATP pyrophosphatase | | - | |  |  |  |  |  |  |  |  |  | |  | |
| 3.5.4.19 | Phosphoribosyl-AMP cyclohydrolase | | - | |  |  |  |  |  |  |  |  |  | |  | |
| 5.3.1.16 | 1-(5-Phosphoribosyl)-5-[(5-phosphoribosylamino)methylideneamino] imidazole-4-carboxamide isomerase | | - | |  |  |  |  |  |  |  |  |  | |  | |
| 2.4.2.- | Pentosyl ribosyltransferase | | - | |  |  |  |  |  |  |  |  |  | |  | |
| 4.2.1.19 | Imidazoleglycerol-phosphate dehydratase | | - | |  |  |  |  |  |  |  |  |  | |  | |
| 2.6.1.19 | Histidinol phosphate aminotransferase | | - | |  |  |  |  |  |  |  |  |  | |  | |
| 3.1.3.15 | Histidinol phosphatase | | - | |  |  |  |  |  |  |  |  |  | |  | |
| 1.1.1.23 | Histidinol dehydrogenase | | - | |  |  |  |  |  |  |  |  |  | |  | |
| **Asparagine biosynthesis** | | | | | | | | | | | | | | | | |
| 6.3.1.1 | Aspartate-ammonia ligase | | *asnA* | |  |  |  |  |  |  |  |  |  | |  | |
| 6.3.5.4 | Asparagine synthetase | | *asnB* | |  |  |  |  |  |  |  |  |  | |  | |

Grey box, gene is present; white box, gene is absent; F, gene fragment(s) only.
