# Supplementary material for: Lactobacillus delbrueckii ssp. lactis and ssp. bulgaricus: a chronicle of evolution in action
Source: BMC Genomics. 2014 May 28;15(1):407. doi: 10.1186/1471-2164-15-407 (PMC4082628; doi:10.1186/1471-2164-15-407)
Supplement: Supplementary file 11 — Additional file 11: Table S7: Genes involved in acid stress resistance. (DOC 54 KB) [file 12864_2014_6193_MOESM11_ESM.doc]

**Add11: Table S7. Genes involved in acid stress resistance.**

|  | | |  | | **ssp*. lactis* strains** | | | | | **ssp. *bulgaricus* strains** | | | | |
| --- | --- | --- | --- | --- | --- | --- | --- | --- | --- | --- | --- | --- | --- | --- |
| EC number | Function | | Gene  Name | CNRZ226 | | CNRZ327 | CNRZ333 | CNRZ700 | NDO2 | ATCC 11842 | ATCC BAA365 | VIB27 | VIB44 | 2038 |
| **Proton pumps and related function** | | | | | | | | | | | | | | |
| 3.6.3.14 | | H+ transporting ATPase / ATP synthase, A subunit | *atpB* | |  |  |  |  |  |  |  |  |  |  |
| 3.6.3.14 | | H+ transporting ATPase / ATP synthase, C subunit | *atpE* | |  |  |  |  |  |  |  |  |  |  |
| 3.6.3.14 | | H+ transporting ATPase / ATP synthase, B subunit | *atpF* | |  |  |  |  |  |  |  |  |  |  |
| 3.6.3.14 | | H+ transporting ATPase / ATP synthase, delta subunit | *atpH* | |  |  |  |  |  |  |  |  |  |  |
| 3.6.3.14 | | H+ transporting ATPase / ATP synthase, alpha subunit | *atpA* | |  |  |  |  |  |  |  |  |  |  |
| 3.6.3.14 | | H+ transporting ATPase / ATP synthase, gamma subunit | *atpG* | |  |  |  |  |  |  |  |  |  |  |
| 3.6.3.14 | | H+ transporting ATPase / ATP synthase, beta subunit | *atpD* | |  |  |  |  |  |  |  |  |  |  |
| 3.6.3.14 | | H+ transporting ATPase / ATP synthase, epsilon subunit | *atpC* | |  |  |  |  |  |  |  |  |  |  |
| **Other functions** | | | | | | | | | | | | | | |
| 4.1.1.15 | | Glutamate decarboxylase | *gadA* | |  | F | F | F | F |  |  |  |  |  |
| 4.1.1.17 | | Ornithine decarboxylase 1 | *-* | |  |  |  |  |  |  |  |  |  |  |
| 4.1.1.17 | | Ornithine decarboxylase 2 | *-* | |  |  |  |  |  |  |  |  |  |  |
| 3.5.3.6 | | Arginine deiminase | *arcA* | |  |  |  | F |  |  |  |  |  |  |
| 2.1.3.3 | | Ornithine carbamoyltransferase | *arcB* | |  |  |  |  |  |  |  |  |  |  |
| 2.7.2.2 | | Carbamate kinase | *arcC* | |  |  |  |  |  |  |  |  |  |  |
| - | | Arginine-ornithine antiporter | *arcD* | |  | F |  |  | F | F | F | F | F | F |

Grey box, gene is present; white box, gene is absent; F, gene fragment(s) only.
